# Localized and Transient Oxygenation of Shallow Oceans of Southwestern Laurentia at the Ediacaran–Cambrian Boundary

**DOI:** 10.1111/gbi.70039

**Published:** 2025-12-30

**Authors:** Watsawan Chanchai, Lyle L. Nelson, Emily F. Smith, Dalton S. Hardisty, Mary C. Lonsdale, Janet E. Burke, Kimberly V. Lau

**Affiliations:** ^1^ Department of Geosciences and Earth and Environmental Systems Institute The Pennsylvania State University University Park Pennsylvania USA; ^2^ Department of Earth and Planetary Sciences Johns Hopkins University Baltimore Maryland USA; ^3^ Department of Earth, Atmosphere and Planetary Sciences Massachusetts Institute of Technology Cambridge Massachusetts USA; ^4^ Department of Earth and Environmental Sciences Michigan State University East Lansing Michigan USA

**Keywords:** BAsal Cambrian carbon isotope excursion (BACE), carbonates, carbonate‐associated uranium isotopes, carbonate‐bound iodine, cerium anomaly, redox proxy, shallow marine environments

## Abstract

The Ediacaran–Cambrian boundary, which precedes one of the most significant biotic diversification events in Earth's history, is associated with a global negative carbon isotope excursion termed the BAsal Cambrian carbon isotope Excursion (BACE). Late Ediacaran and early Cambrian changes in shallow marine oxygenation have been proposed to relate to the BACE as well as metazoan extinction and radiation. However, reconstructing paleoredox conditions at the Ediacaran–Cambrian boundary is limited by challenges in correlating carbonate strata due to sparse stratigraphic markers and non‐unique chemostratigraphic correlations. These imprecise correlations have led to uncertainty in how redox changes across the BACE should be interpreted in relation to broader regional and global environmental patterns. Here, we present redox reconstructions from southwestern Laurentian carbonate successions that record the BACE, including the limestone‐dominated Deep Spring Formation, southwestern USA, and the dolostone‐dominated La Ciénega Formation, northern Mexico. We combine local (carbonate‐bound iodine, I/(Ca + Mg) and cerium anomaly, Ce/Ce*) and global (carbonate‐associated uranium isotopes, δ^238^U_carb_) redox proxies to investigate marine oxygenation in relation to the BACE. Contrary to previous suggestions that a global ocean oxygenation event coincided with the BACE, we do not observe a shift in δ^238^U_carb_ concurrent with the carbon isotope excursion in either section. The δ^238^U_carb_ values differ between two sections, likely reflecting distinct diagenetic offsets attributed to different diagenetic U reduction, but together provide a minimal constraint on the carbonate δ^238^U value and suggest a more anoxic ocean compared to today. The local proxy results at both sites suggest widespread low‐oxygen surface waters with a transient and localized interval of shallow marine oxygenation at one site that coincides with the nadir of the BACE. Persistently low I/(Ca + Mg) ratios, below values observed in today's oxygenated oceans, suggest a broadly redox‐stratified surface ocean. Negative Ce anomalies in the La Ciénega Formation were recorded during the BACE nadir, suggesting a short‐lived interval of local oxygenation within otherwise low‐oxygen conditions. In sum, we do not find evidence for major, widespread oxygenation coincident with the BACE, but a continuation of low‐oxygen conditions punctuated by a short‐lived oxygenation event in the shallow oceans. These brief fluctuations in oxygen levels, in turn, may have played a role in the onset of behavioral complexity among bilaterian invertebrates during this critical transition.

## 1 Introduction

The Ediacaran–Cambrian transition (ca. 550–533 Ma) was a period marked by significant evolutionary and ecological changes (e.g., Darroch et al. 2018, 2023; Mángano and Buatois 2016; Seilacher et al. 2005; Wood et al. 2019; Zhu et al. 2017; Zhuravlev and Wood 2018). During this period, in addition to the advent of diverse animal body plans, body and trace fossil records indicate significant ecological developments, including the emergence of ecosystem engineering behaviors such as intensive and complex bioturbation, and ecological interactions like predation (Darroch et al. 2018, 2023; Mángano et al. 2012; Sperling et al. 2013). A long‐standing debate centers on whether this biotic revolution was a consequence of environmental changes, specifically an increase in atmospheric oxygen levels in the late Neoproterozoic that may have driven the rise of early animals (e.g., Cole, Mills, et al. 2020; Lyons et al. 2014; Och and Shields‐Zhou 2012; Shi et al. 2022; Stockey et al. 2024). Based on Cr isotope records and other constraints on redox conditions during this time, atmospheric oxygen is thought to be > 0.1%–1% present atmospheric level (PAL) (e.g., Crockford et al. 2018; Farquhar et al. 2000; Hardisty and Lau 2024; Kump 2008; Lyons et al. 2014; Planavsky et al. 2014; Sperling, Knoll, and Girguis 2015). At these low levels of atmospheric oxygen, the distribution of dissolved oxygen in the oceans is expected to be heterogenous in shallow marine settings (Reinhard et al. 2016; Stockey et al. 2024). Sufficient oxygenation of shallow‐water Ediacaran and Cambrian environments may be a requirement for complex metazoan behaviors and ecological interactions (Evans et al. 2018, 2022; Sperling, Knoll, and Girguis 2015; Sperling et al. 2022). Therefore, reconstructing shallow marine redox conditions is important for understanding if and how environmental changes impacted early metazoan radiation (Wood et al. 2019; Wood and Erwin 2018). However, determining the relationship between redox conditions and evolution is challenging. Among many reasons, this is difficult because many constraints on ocean oxygenation during the late Neoproterozoic come from deep‐water shales (e.g., Canfield et al. 2007; Poulton and Canfield 2011; Sperling, Knoll, and Girguis 2015; Wood et al. 2015), which may not directly reflect early animal habitats in shallow shelf environments. Spatially constraining shallow oxygenation requires multiple proxies and sites to establish a comprehensive global picture of paleoenvironmental changes and to assess the effects of diagenetic alteration on carbonate geochemistry.

**FIGURE 1:**
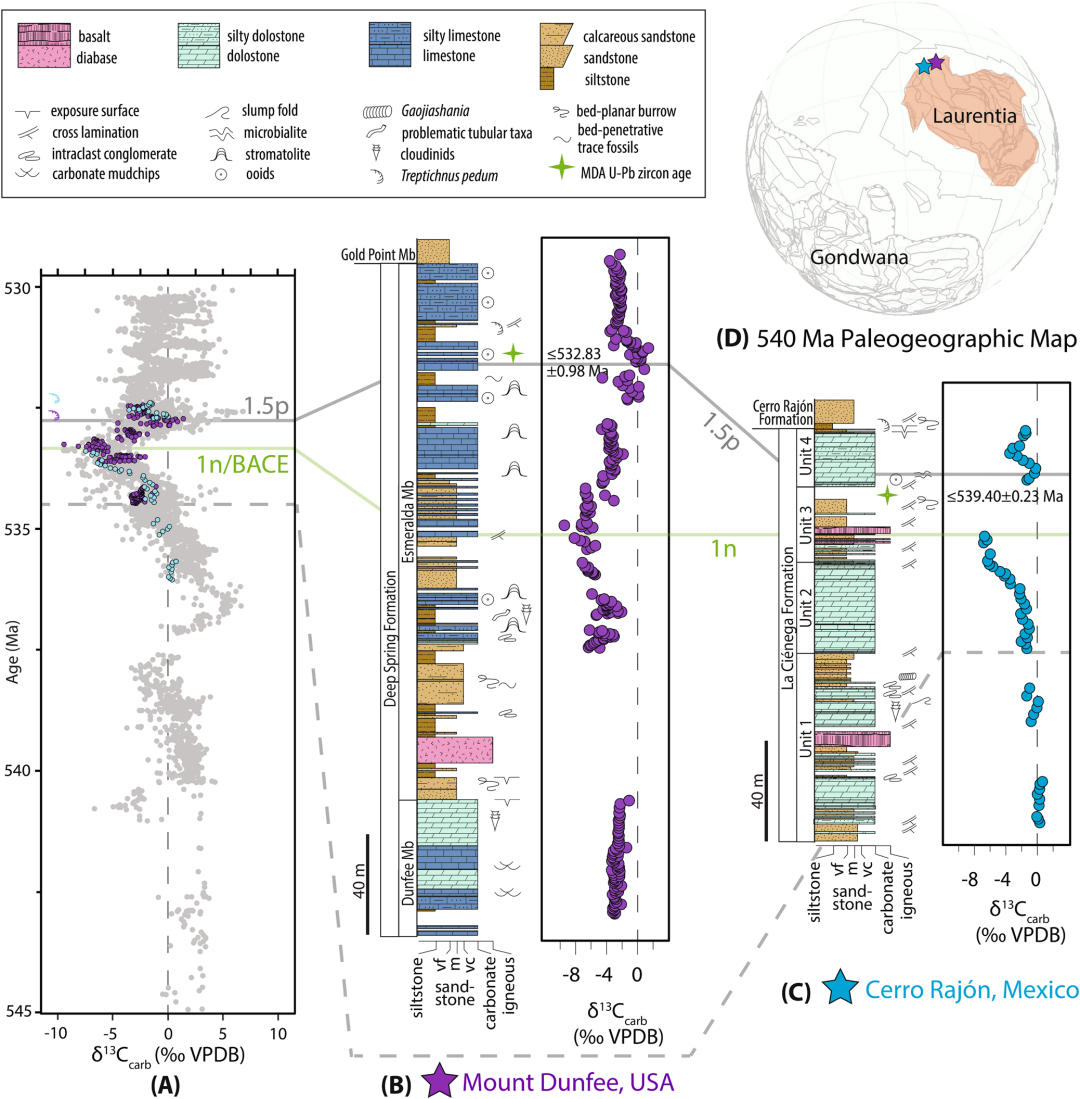
(A) Compilation of carbonate δ^13^C data from 545 to 530 Ma with radiometric and fossil occurrence age constraints (after model age K in Bowyer et al. 2022; Bowyer, Uahengo, et al. 2023) that follows the age model of Nelson et al. (2023). (B–C) Lithostratigraphy and δ^13^C chemostratigraphy for (B) Mount Dunfee, USA (after Smith, Nelson, et al. 2016) and (C) Cerro Rajón, Mexico (after Hodgin et al. 2021). The dashed gray line, green line labeled “1n”, and gray line labeled “1.5p” correspond to the approximate onset, nadir, and recovery of the BACE, respectively, based on chemostratigraphic correlations from Nelson et al. (2023). The two radioisotopic ages are U–Pb zircon CA‐ID‐TIMS ages that represent maximum depositional age (MDA) constraints in the Cerro Rajón section (Hodgin et al. 2021) and Mount Dunfee section (Nelson et al. 2023). (D) The 540 Ma paleogeographic map shows studied sites, marked by stars (after Merdith et al. 2021). VPDB–Vienna‐Pee Dee Belemnite; vf–very fine‐grained, m–medium‐grained, vc–very coarse‐grained.

Reconstructing regional and global redox changes in shallow marine environments requires sampling from coeval carbonate‐dominated paleogeographic locations. However, identifying precisely correlative carbonate successions that span the Ediacaran–Cambrian boundary is challenging due to (1) taphonomic biases of biostratigraphically significant body and trace fossils, (2) scarcity of radiometric age constraints across Ediacaran–Cambrian transition strata, and (3) non‐unique carbon isotope chemostratigraphic correlations (Bowyer et al. 2022; Bowyer, Uahengo, et al. 2023; Nelson et al. 2023). The base of the Cambrian Period is defined by the first appearance datum (FAD) of *Treptichnus pedum* at Fortune Head, Newfoundland (Brasier et al. 1994; Landing 1994). However, *T. pedum* is not generally found in carbonate facies due to taphonomic biases and/or an ecological preference for siliciclastic substrates (Babcock et al. 2014; Gehling et al. 2001; Geyer 2005), making the identification of the boundary difficult in carbonate‐dominated successions. The BAsal Cambrian carbon isotope Excursion (BACE) is a negative carbon isotopic (δ^13^C) excursion that is commonly used as a secondary marker of the Ediacaran–Cambrian boundary (e.g., Bowyer et al. 2022; Bowyer, Uahengo, et al. 2023; Landing et al. 2007; Nelson et al. 2023; Zhu et al. 2006). The BACE was first documented just below the first local occurrence of *T. pedum* in western North America (Corsetti and Hagadorn 2000; Narbonne et al. 1994), and since then, has been identified in multiple carbonate‐rich sections worldwide and is now considered a global chemostratigraphic marker (e.g., Amthor et al. 2003; Bowyer et al. 2022; Bowyer, Zhuravlev, et al. 2023; Brasier et al. 1990, 1996; Corsetti and Hagadorn 2003; Hodgin et al. 2021; Kaufman et al. 1996; Maloof et al. 2010; Nelson et al. 2023; Smith, Macdonald, et al. 2016; Smith, Nelson, et al. 2016; Smith et al. 2023; Topper et al. 2022; Yang et al. 2021, 2023; Zhang et al. 2024; Zhu et al. 2017; Zhu et al. 2006). A recent global carbon isotope compilation (Bowyer et al. 2022, 2024) places the falling limb of the BACE at ~536 Ma and the nadir of the excursion at ~534 Ma, with recovery of δ^13^C values by ~532 Ma (Figure 1A).

The BACE broadly coincides with the disappearance of the Ediacaran biota and the appearance of complex bilaterian trace fossils and early skeletonized animals. This negative δ^13^C excursion, which reaches carbonate δ^13^C values of < −7‰, is also hypothesized to represent a global carbon cycle perturbation (Darroch et al. 2018, 2023). Negative δ^13^C excursions can result from multiple drivers, such as decreased organic carbon burial caused by a more oxygenated ocean, declines in primary productivity, or via increased input of isotopically depleted carbon reservoirs such as mantle carbon, exogenous organic carbon, and/or methane (e.g., Berner 2004; Broecker 1970; Kump and Arthur 1999). In addition, carbonate δ^13^C values can reflect local and/or post‐depositional controls, such as the proportions of distinct carbonate minerals, alteration from diagenetic fluids, and heterogeneity in seawater δ^13^C values (Ahm et al. 2018; Dyer et al. 2017; Fantle et al. 2020; Geyman and Maloof 2021; Hodgin et al. 2021; Hoffman and Lamothe 2019; Jiang et al. 2012; Stewart et al. 1984). Negative δ^13^C excursions can also reflect higher contributions of authigenic carbonate precipitation, which can occur independently of global ocean oxygenation (Laakso and Schrag 2017; Schrag et al. 2013). Bowyer et al. (2024) observed that, on many paleocontinents, the falling limb of the BACE (~536–534 Ma) is broadly associated with higher carbonate uranium isotope values and transgressive stratigraphic sequences. They propose that global eustatic changes may be linked to shallow marine oxygenation, the BACE, and early Cambrian animal radiation. Major transgressions can expand the area of habitable shelf and enhance nutrient delivery, while the shoaling of anoxic seawater could promote organic carbon burial, leading to increased atmospheric and oceanic oxygen levels. However, the redox proxy records compiled in Bowyer et al. (2024) are from sections in Morocco, Siberia, and South China that have limited age constraints and do not preserve a complete record of the BACE (Cherry et al. 2022; Dahl et al. 2019; Wei et al. 2018; Zhang, Xiao, et al. 2018).

Although the majority of redox proxy records indicate Ediacaran–Cambrian oceans were much more anoxic than today, data from individual sites indicate dynamic redox variations in both deep‐ and shallow‐ water settings. The oceans were redox stratified, and deep waters were broadly ferruginous during the Neoproterozoic and early Paleozoic (Johnston et al. 2010; Li et al. 2010; Lu et al. 2018; Poulton and Canfield 2011; Sperling, Wolock, et al. 2015). Multiple short‐lived episodes of increased oceanic oxygenation are inferred from redox‐sensitive elements in shale and carbonate‐based redox proxies, representative of deep‐ and shallow‐ water settings, respectively (e.g., Bowyer et al. 2022, 2024; Hardisty and Lau 2024; Sahoo et al. 2016; Tostevin and Mills 2020; Wood et al. 2019; Zhang et al. 2019). Carbonate‐based uranium isotope data have been used to suggest globally persistent and widespread anoxic bottom waters during Ediacaran–Cambrian time (Cherry et al. 2022; Kendall et al. 2015; Tostevin, Wood, et al. 2016; Wei et al. 2018; Wei, Planavsky, et al. 2020; Zhang, Xiao, et al. 2018; Zhang et al. 2019; Zhang et al. 2019). Statistical analysis of a compilation of local Fe speciation data suggests a broadly ferruginous Neoproterozoic deep ocean, with no significant increase in oxygenation into the Cambrian (Sperling, Wolock, et al. 2015; Stockey et al. 2024). However, this reconstruction of anoxic conditions is in contrast to the interpretations drawn from molybdenum isotope and site‐specific Fe speciation records from Newfoundland, Russia, and Brazil that suggest that oceans were becoming progressively more oxygenated during the early Cambrian (Canfield et al. 2007; Caxito et al. 2024; Johnston et al. 2012). Carbonate‐based redox proxies also suggest transient, localized oxygenation of surface seawater in parts of the Nama Basin, Namibia during the latest Ediacaran (Bowyer et al. 2020; Tostevin, Wood, et al. 2016; Uahengo et al. 2020; Wood et al. 2015). This discrepancy in whether oceans were anoxic or becoming oxygenated at the Ediacaran–Cambrian boundary could reflect the global versus local nature of specific proxies, as well as if sites represent shallow versus deep marine environments. The potential for dynamic redox conditions, against the backdrop of an anoxic ocean, may be linked to carbon isotope excursions and associated ecological and environmental shifts across the Ediacaran–Cambrian transition.

Both global and local redox conditions during the BACE and their potential link(s) to the carbon cycle are not well understood. Whether redox conditions can be decoupled from carbon isotope variability (Ostrander 2023; Ostrander et al. 2023), including the BACE, remains an outstanding question. In this study, we compare a suite of local and global redox proxies—carbonate‐bound iodine (I/(Ca + Mg)), cerium anomaly (Ce/Ce*), and uranium isotopes (δ^238^U_carb_)—from two correlative Ediacaran–Cambrian carbonate sections in southwestern Laurentia (southwestern USA and northern Mexico). Our multi‐proxy approach allows us to reconstruct shallow‐water redox landscapes within a constrained age framework. These records reveal the stability, synchronicity, and direction of redox changes across the Ediacaran–Cambrian boundary and how these changes relate to the BACE. Ultimately, this study aims to improve the understanding of the relationship between redox change, carbon isotope excursion, and paleoenvironmental perturbations during this critical evolutionary transition.

## 2 Background

### 2.1 Geologic Setting

The Deep Spring Formation at Mount Dunfee (Nevada, USA) and the La Ciénega Formation at Cerro Rajón (Sonora, Mexico) have been previously correlated using biostratigraphy, carbon isotope chemostratigraphy, and radioisotopic geochronology (Hodgin et al. 2021; Nelson et al. 2023; Smith, Nelson, et al. 2016; Smith et al. 2023). Both of these sections record the BACE, preserve the trace fossil *T. pedum*, and span the Ediacaran–Cambrian boundary (Figure 1). Today, these sections are separated by ~1000 km, although there is debate on the palinspastic structural reconstruction of this margin, and the Caborca Block of Sonora may restore to the northwest, more proximal to Nevada.

#### 2.1.1 Mount Dunfee, Nevada, USA

Upper Ediacaran to lower Cambrian strata in the Great Basin of the southwestern United States were deposited during the transition from a rifted margin to a passive margin during the breakup of Rodinia (Fedo and Cooper 2001; Nelson 1978; Smith et al. 2023; Stewart 1970). In the White‐Inyo Ranges (eastern California) and in Esmeralda County (western Nevada), Ediacaran–Cambrian boundary strata comprise the Deep Spring Formation (Ahn et al. 2011; Albers and Stewart 1972; Corsetti and Hagadorn 2003; Smith, Nelson, et al. 2016; Smith et al. 2023). At Mount Dunfee in Nevada, the Deep Spring Formation is ~455 m thick and is composed of interbedded limestone and dolostone, siltstone, calcareous sandstone, and quartzite (Ahn et al. 2011; Gevirtzman and Mount 1986; Signor et al. 1987; Smith, Nelson, et al. 2016; Stewart 1970) (Figure 1B). The Deep Spring Formation is divided into three members: the Dunfee, Esmeralda, and Gold Point members. The samples examined in this study are from the upper Dunfee Member and the Esmeralda Member.

The Dunfee Member consists of limestone and recrystallized dolostone. The contact with the overlying Esmeralda Member is marked by a karst surface that is overlain by sandstone with mud cracks, indicative of subaerial exposure (Smith, Nelson, et al. 2016). The Esmeralda Member consists of mixed sandstone, mudstone, and limestone (Albers and Stewart 1972; Smith, Nelson, et al. 2016). The Deep Spring Formation contains a diverse faunal assemblage, including pyritized and calcified cloudinomorphs, other tubular body fossils, such as *Gaojiashania* and *Wutubus annularis*, and trace fossils including *Bergaueria*, treptichnids, and simple bed‐planar structures (Grant 1990; Selly et al. 2020; Signor et al. 1987; Smith, Nelson, et al. 2016; Tarhan et al. 2020). The sedimentary facies found throughout the Deep Spring Formation have been used to suggest that this unit was deposited in a shallow marine environment, ranging from peritidal to shoreface (Oliver and Rowland 2002; Rowland et al. 2008; Smith, Nelson, et al. 2016; Tarhan et al. 2020).

The BACE is identified in multiple sections across the Great Basin. At Mount Dunfee, the δ^13^C values decrease to below ~−6‰, and the lowest local occurrence of *T. pedum* is within the recovery of the δ^13^C excursion (Corsetti and Hagadorn 2003; Smith, Nelson, et al. 2016; Smith et al. 2023) (Figure 1B). Based on regional correlations to the lower member of the Wood Canyon Formation in southern Nevada, the recovery of the BACE (i.e., 1.5p excursion) and the lowest occurrence of *T. pedum* in the Mount Dunfee section is constrained to ≤ 532.83 ± 0.98 Ma, a maximum depositional age from zircon U–Pb dates (Nelson et al. 2023).

#### 2.1.2 Cerro Rajón, Sonora, Mexico

The Caborca region in Sonora, Mexico, preserves upper Ediacaran and lower Cambrian shallow marine strata that are correlative to those found in the southern Great Basin (Stewart et al. 1984). In this region, the Ediacaran–Cambrian transition is recorded in the La Ciénega and Cerro Rajón formations (Barrón‐Díaz et al. 2019; Hodgin et al. 2021; Sour‐Tovar et al. 2007). The La Ciénega Formation is approximately 270 m thick at the Cerro Rajón section, comprising four intervals, dominated by dolo‐grainstone and recrystallized dolostone and separated by intervals of sandstone and mudstone with subordinate dolostone and basalt, which define the four informal units (Hodgin et al. 2021; Stewart et al. 1984) (Figure 1C). The La Ciénega Formation is overlain by the Cerro Rajón Formation which contains sandstone, conglomerate, volcaniclastics, basalt, and minor limestone (Barrón‐Díaz et al. 2019). In addition to marine sedimentary strata, the La Ciénega Formation contains several horizons of basalt in the upper parts of Unit 1 and Unit 3 that have been interpreted as shallow early Cambrian sills (Tapia‐Trinidad et al. 2022). A prevalence of cross‐stratified grainstone and occurrences of marine fossils, ooids, and stromatolites have been used to suggest that the La Ciénega Formation was deposited on a shallow marine carbonate platform (Hodgin et al. 2021).

The La Ciénega Formation contains an assemblage of dolomitized and silicified late Ediacaran tubular fossils, including *Cloudina*, *Saarina*, and *Sinotubulites* (Schiffbauer et al. 2024; Sour‐Tovar et al. 2007), and overlying sandstone and siltstone preserve casts and molds of the annulated tubular fossils c.f. *Gaojiashania* (Hodgin et al. 2021). All of these tubular taxa are considered potential late Ediacaran index fossils. The overlying Cerro Rajón Formation contains complex bed‐penetrating bilaterian ichnotaxa, including *T. pedum* and subvertical to vertical burrows c.f. *Skolithos* (Barrón‐Díaz et al. 2019; Hodgin et al. 2021; Sour‐Tovar et al. 2007), indicative of deposition in the early Cambrian Period.

Dolostones in Unit 1 of the La Ciénega Formation contain carbonate δ^13^C values of ~0‰, and units 2 and 3 record a decline to δ^13^C values < −6‰, which has been correlated to the BACE (Hodgin et al. 2021; Loyd et al. 2012) (Figure 1C). In Unit 4, the recovery of the BACE is recorded by the return of δ^13^C values to ~0‰, which can be correlated to regional chemostratigraphic records (i.e., 1.5p excursion across the southern Great Basin (Nelson et al. 2023)). The δ^13^C trends of the La Ciénega Formation are reproducible in several sections in the Caborca region, as well as within correlated carbonate‐rich sections in Nevada and California (Hodgin et al. 2021; Nelson et al. 2023; Smith et al. 2023).

#### 2.1.3 Stratigraphic Correlations

Upper Ediacaran and lower Cambrian strata in this study are correlated based on previously established age models based on lithostratigraphy, paleontology, δ^13^C chemostratigraphy, and U–Pb geochronology (e.g., Hodgin et al. 2021; Smith et al. 2023; Stewart 1974). There are two radioisotopic age constraints of the BACE within the Ediacaran–Cambrian strata in southwest North America. One is from a sandy dolostone bed above the nadir of the BACE in Unit 3 of the La Ciénega Formation. Here, U–Pb dates obtained by chemical abrasion–isotope dilution–thermal ionization mass spectrometry (CA‐ID‐TIMS) on detrital zircons were reported as 539.40 ± 0.23 Ma; this date was interpreted as a maximum depositional age for the BACE (Hodgin et al. 2021). Additional radioisotopic age data are from the lower member of the Wood Canyon Formation in Nevada. Detrital zircons found in beds intercalated with strata recording the recovery of the BACE were also dated using CA‐ID‐TIMS. These data were used to suggest that the recovery of the BACE (i.e., 1.5p) has a maximum depositional age of 532.83 ± 0.98 Ma (Nelson et al. 2023). Together, these constraints suggest Unit 3 of the La Ciénega Formation is likely several million years younger than the youngest zircon grains dated from this unit. These age constraints on the BACE from Nevada and Mexico have been integrated into a global age model that uses carbonate δ^13^C chemostratigraphy, biostratigraphy, and other radioisotopic data to constrain the BACE to an interval from ~536 Ma to 532 Ma (Bowyer et al. 2022, 2024; Nelson et al. 2023). This age framework places the BACE younger than the official age of the Ediacaran–Cambrian boundary but serves as a useful reference to compare our redox data with previous studies.

#### 2.1.4 Calcium Isotope Constraints on Diagenesis

Calcium isotopes (δ^44/40^Ca) have been used to evaluate the extent and style of diagenesis in carbonate rocks, with higher δ^44/40^Ca values interpreted to represent a greater extent of diagenesis (Ahm et al. 2018; Gussone et al. 2020; Husson et al. 2015; Lau, Maher, et al. 2017). To assess diagenesis, Ca isotopes are commonly compared with Sr/(Ca + Mg) ratios, which tend to decrease with greater diagenetic alteration. In the La Ciénega Formation at Cerro Rajón, Sr/(Ca + Mg) ratios are consistently low and δ^44/40^Ca data reach maximum values of −0.68‰ (Lonsdale 2025; Lonsdale et al., n.d.). At Mount Dunfee, Sr/(Ca + Mg) ratios are higher and more variable, with δ^44/40^Ca values reaching maximum values of −0.95‰ and −0.69‰ in the upper Dunfee Member and Esmeralda Member of the Deep Spring Formation, respectively (Lonsdale 2025; Lonsdale et al., n.d.). The low Sr/(Ca + Mg) ratios and high δ^44/40^Ca values of BACE‐bearing strata in the Esmeralda Member and La Ciénega Formation have been interpreted to broadly record seawater‐buffered conditions (with respect to Ca) during early marine diagenesis. Conversely, higher Sr/(Ca + Mg) ratios and lower δ^44/40^Ca values in the Dunfee Member are interpreted to reflect more sediment‐buffered conditions (with respect to Ca) during early marine diagenesis, despite increasing Mg/Ca ratios associated with dolomitization in the upper Dunfee Member. These data are interpreted to suggest limited alteration the initial carbonate δ^13^C values (Lonsdale 2025; Lonsdale et al., n.d.), lending support for the potential to preserve seawater redox signals.

### 2.2 Carbonate Paleoredox Proxies

**FIGURE 2:**
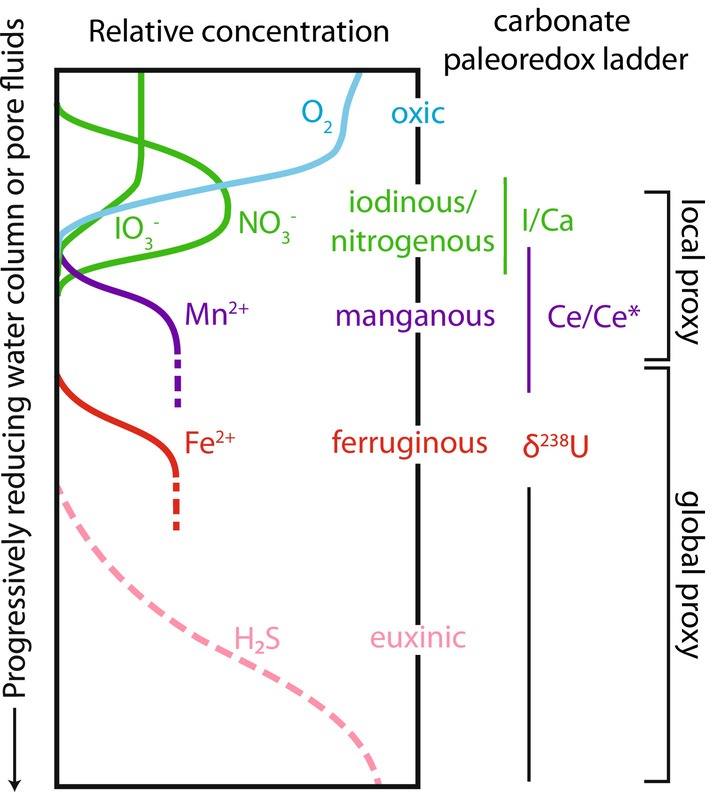
The carbonate paleoredox “ladder” depicting the redox potential of the oxidized species for the redox proxies in this study (after Lau and Hardisty 2022).

We used three carbonate‐based redox proxies to reconstruct marine redox conditions. Each of these proxies has a distinct redox potential, residence time, sensitivity to local versus global redox conditions (Figure 2), and susceptibility to diagenetic alteration (e.g., Ahm et al. 2018; Lau and Hardisty 2022). Therefore, the fidelity of each proxy should be evaluated independently prior to interpretations of redox conditions.

#### 2.2.1 Global Redox Proxy: Uranium Isotopes

Uranium isotope ratios (^238^U/^235^U, denoted in standard delta notation as δ^238^U relative to CRM‐145) in carbonates have been used to track global marine redox changes (Chen, Tissot, et al. 2021; Lau et al. 2019; Zhang et al. 2019). In the modern ocean, uranium has a long residence time (approximately 400 kyr), significantly longer than the ocean mixing time (Dunk et al. 2002; Ku et al. 1977). The proxy is based on the observation that uranium reduction, which thermodynamically is predicted to occur at the Fe^3+^/Fe^2+^ redox potential, is associated with mass‐independent isotope fractionation (e.g., Mioduski 1999; Schauble 2007) (Figure 2). The average δ^238^U of modern seawater is −0.379‰ ± 0.023‰ (Kipp et al. 2022; Tissot and Dauphas 2015) and is lower than the average δ^238^U of continental crust of −0.29‰ ± 0.03‰ (Chen, Tissot, et al. 2021; Li and Tissot 2023). In natural settings, ^238^U is preferentially incorporated in sparingly soluble U (IV), which is then sequestered in reducing environments (Stirling et al. 2007; Weyer et al. 2008). This process leaves seawater U (VI) with a lower δ^238^U value. Uranium reduction and associated isotope fractionation in sediments are diffusion‐limited, with the magnitude of the isotope offset dependent on the degree of reducing conditions and organic carbon loading (Andersen et al. 2014; Bruggmann et al. 2022; Lau et al. 2020; Rutledge et al. 2024). In the modern ocean, U reduction is observed in sulfate reducing settings, and therefore it has been proposed that the δ^238^U proxy best tracks sulfidic conditions (Brüske et al. 2020; Cole, Planavsky, et al. 2020; Gilleaudeau et al. 2019; Stockey et al. 2020). However, U reduction is not necessarily limited to sulfidic conditions during other periods of Earth history when other biogeochemical reactions were more dominant (e.g., Gilleaudeau et al. 2019; Lau et al. 2022; Zhang, Lenton, et al. 2020). Therefore, in this study we interpret the δ^238^U proxy to reflect changes in anoxia, and not specifically sulfidic conditions. In sum, anoxic sediments have higher δ^238^U values compared to seawater, and during times of greater anoxia, the expansion of the area of anoxic sediments results in lower seawater δ^238^U values (Zhang, Lenton, et al. 2020).

Carbonate‐associated δ^238^U_carb_ is thought to track seawater δ^238^U composition but with an isotope offset. Based on measurements of the δ^238^U_carb_ in Neogene carbonate samples, bulk carbonates exhibit a positive offset of ~0.27‰ with a large range (0‰ to +0.5‰), relative to seawater (Chen et al. 2018; Romaniello et al. 2013; Tissot et al. 2018). This offset is argued to reflect early diagenetic reduction of uranium and incorporation of ^238^U‐enriched U (IV) in recrystallized carbonates or diagenetic cements (Chen et al. 2018; Tissot et al. 2018; Yuan et al. 2023). Modeling indicates that early marine diagenesis with reducing seawater has the potential to produce large positive offsets in δ^238^U_carb_, whereas the effects of meteoric diagenesis are limited due to typically low uranium concentrations in meteoric fluids (Lau and Hardisty 2022). Based on studies from Bahamas sediments and Jurassic platform carbonates, dolomitization is not expected to impart an additional offset between the δ^238^U of the carbonate and seawater (Chen et al. 2018; Herrmann et al. 2018). However, the effects of dolomitization on δ^238^U_carb_ values can vary widely depending on the specific diagenetic conditions for a given set of samples.

#### 2.2.2 Local Redox Proxies: Carbonate‐Bound Iodine and Cerium Anomaly

In the modern ocean, iodate is reduced in the iodinous zone, whereas cerium is reduced in the manganous zone (Cutter et al. 2018; Moriyasu et al. 2020; Rue et al. 1997). In other words, the I/(Ca + Mg) proxy is expected to be more sensitive to oxygen availability than the Ce anomaly despite their overlapping redox gradient (Figure 2). Therefore, seawater signals recorded by the Ce anomaly and I/(Ca + Mg) ratio could represent geographic and temporal differences between iodinous and manganous conditions.

Iodine (I) is a micronutrient whose speciation in seawater is sensitive to surface‐water redox conditions. Iodate (IO_3_^−^) is reduced to iodide (I^−^) in anoxic seawater and reducing pore fluids at broadly nitrogenous conditions (i.e., iodinous) (Cutter et al. 2018; Hardisty et al. 2020; Moriyasu et al. 2020; Rue et al. 1997) (Figure 2). Therefore, iodate levels are particularly suitable for tracking redox conditions relevant for early metazoans that are thought to be sensitive to redox potentials equivalent to hypoxic ocean regions today (Wei et al. 2019; Wörndle et al. 2019). Iodate is the only iodine species that is incorporated into the crystal lattice of aragonite, calcite, and dolomite minerals and therefore its abundance can be reported as carbonate‐bound iodine ratios (I/(Ca + Mg)) in carbonate rocks (Hashim et al. 2022; Lu et al. 2010; Podder et al. 2017). In modern calcitic foraminifera samples, I/(Ca + Mg) ratios of < 2.6 μmol/mol occur exclusively in oxygen‐deficient waters (Glock et al. 2014; Hardisty et al. 2014, 2017; Lu et al. 2018; Lu et al. 2010; Shang et al. 2019; Zhou et al. 2014). A similar ratio is expected to apply to inorganic carbonates, since I/(Ca + Mg) ratios below this threshold correspond to iodate concentrations found in modern oxygen‐deficient zones (Hardisty et al. 2021). These low I/(Ca + Mg) ratios can indicate reducing (iodinous) conditions in shallow surface waters or a more broadly redox‐stratified ocean because oxidation to iodate is kinetically limited, even in oxic surface waters (Glock et al. 2014; Hardisty et al. 2021; Lu et al. 2016). In addition, zero I/(Ca + Mg) ratios (i.e., below detection) can indicate either fully anoxic conditions and/or diagenetic alteration due to iodate removal from the carbonate lattice during recrystallization in reducing pore fluids, which has been shown to always result in lower I/(Ca + Mg) ratios (Hardisty et al. 2017; Lau and Hardisty 2022). To differentiate redox conditions from diagenetic signals, I/(Ca + Mg) records should be interpreted alongside stratigraphic and lithologic constraints (Hardisty et al. 2017).

Rare earth elements and yttrium (REY), specifically the relative abundance of cerium (Ce) to its neighboring elements, are an established redox proxy in carbonate rocks (Tostevin 2021; Wallace et al. 2017; Zhao et al. 2021). The cerium anomaly (Ce/Ce*) is a robust tool for constraining water‐column redox conditions because Ce cycling is associated with the reduction of manganese oxides, which occurs at a redox potential between iodate reduction and uranium reduction (i.e., manganous zone) (Lawrence and Kamber 2006) (Figure 2). Whereas other REY are present only in a 3+ oxidation state at surface conditions, cerium oxidizes from a soluble trivalent form, Ce (III), to an insoluble tetravalent form, Ce (IV), in oxygenated seawater. The Ce (IV) is adsorbed to Mn‐ and Fe‐ oxides and scavenged, resulting in a negative cerium anomaly (Ce/Ce*) in oxygenated seawater and carbonate rock records through carbonate complexation (Bau and Koschinsky 2009; de Baar et al. 1985; Elderfield 1988). The Ce anomaly is calculated by comparing Ce concentration to its neighboring REY normalized to Post Archean Australian Shale (PAAS), denoted as *SN*: $\mathrm{Ce}/{\mathrm{Ce}}^{*}=\frac{{\mathrm{Ce}}_{\mathrm{SN}}}{{\left({\mathrm{Pr}}_{\mathrm{SN}}\right)}^{2}/{\mathrm{Nd}}_{\mathrm{SN}}}$ (Lawrence et al. 2006; Pourmand et al. 2012). A negative anomaly indicative of oxic conditions is defined using a threshold of Ce/Ce* < 0.8 and a positive anomaly indicative of anoxic conditions is defined using a threshold of Ce/Ce* > 1.2 based on modern manganous waters (Ce/Ce* values of 1.21–2.43) and carbonate records (Bau et al. 1997; de Baar et al. 1985; de Carlo and Green 2002; Tostevin, Wood, et al. 2016; Tostevin 2021; Wallace et al. 2017). If Ce/Ce* values fall in between the conservative redox thresholds (i.e., 0.8 < Ce/Ce* < 1.2) (Tostevin 2021), the lack of Ce anomalies is considered to reflect seawater that is not strongly oxic nor anoxic.

Compared to other carbonate paleoredox proxies, Ce/Ce* is considered to be robust to early diagenetic alteration, due to limited Ce/Ce* value changes during diagenesis (Banner et al. 1988; Lau and Hardisty 2022; Liu et al. 2019; Webb et al. 2009). The effect of remobilization and post‐depositional alteration on Ce anomalies can be assessed by evaluating the REY distribution and middle rare earth element (MREE) enrichment that reflect the distinct patterns of different source fluids (Shields and Stille 2001; Zhang and Shields 2023; Zhao et al. 2022). In the upper ocean today, light rare earth elements (LREE) are preferentially scavenged compared to the heavy rare earth elements (HREE) due to shorter scavenging residence time of LREE during carbonate complexation and LREE preferential removal during remineralization of surface coating on particles at depth (Byrne and Kim 1990; Cantrell and Byrne 1987; Elderfield 1988; Schijf et al. 2015; Sholkovitz et al. 1994), producing a seawater‐like REY pattern enriched in HREE. If carbonate rocks do not contain this expected REY pattern, it could indicate the influence of a non‐seawater source. Yttrium (Y) is not a lanthanide, but it behaves similarly to holmium (Ho) in aqueous solution due to their identical ionic charge and crystallographic ionic radii (Bau et al. 1997; Möller 2022), and therefore it is commonly included as a tracer of REE behavior. Carbonates that record seawater chemistry should contain Y/Ho ratios > 36 (Bau et al. 1996; Liu et al. 2019; Tostevin 2021). Carbonate samples with Y/Ho < 36 may indicate contributions from non‐carbonate phases, such as Fe‐Mn (oxyhydr)oxide and/or clay minerals (Bau et al. 1996; Frimmel 2009; Nothdurft et al. 2004; Taylor and McLennan 1985). Hydrothermal fluids also produce distinctive REY patterns because europium (Eu) is concentrated in these fluids compared to other sources, which can then be enriched in sediments through hydrothermal metalliferous precipitation or magmatism‐influenced diagenesis (MacRae et al. 1992). This results in a large positive Eu anomaly (Eu/Eu*, calculated as: $\mathrm{Eu}/{\mathrm{Eu}}^{*}=\frac{Eu_{\mathrm{SN}}}{{\left({\mathrm{Sm}}_{\mathrm{SN}}^{2}*{\mathrm{Tb}}_{\mathrm{SN}}\right)}^{\frac{1}{3}}}$) (Lawrence et al. 2006). The REY patterns of modern seawater exhibit variable but small positive Eu anomalies (~1.5) (Tostevin, Shields, et al. 2016) due to systematic Eu scavenging by hydroxide minerals (Mitra et al. 1994; Olivarez and Owen 1991). In modern anoxic seawater, the Eu anomaly is not a reliable indicator of basinal anoxia, because these environments can be characterized by both positive Eu/Eu* or no Eu anomaly (e.g., de Baar et al. 1988; Schijf et al. 1995).

## 3 Methods

We selected 50 carbonate samples (22 from Mount Dunfee and 28 from Cerro Rajón) that were previously analyzed for carbon and oxygen isotopes; carbonate content; and major and trace element concentrations (Hodgin et al. 2021; Smith, Nelson, et al. 2016). The samples were trimmed and powdered using a Dremel with a diamond bit or with a ball mill in a zirconia ceramic grinding vial. Samples with high carbonate content (> 90 *wt*.%) were selected for paleoredox proxy analyses. We screened for secondary alteration using petrography, oxygen isotope, and elemental ratios. Nine representative thin sections were selected for stratigraphic coverage to observe carbonate fabrics, mineralogy, and diagenetic features (see Data S1 for full details). All sample dissolution and preparation steps were performed in a metal‐free clean laboratory at Pennsylvania State University.

### 3.1 Uranium Isotopes

Sufficient carbonate powder was dissolved to obtain 300 ng U for isotopic analysis. Following methods from Clarkson et al. (2020), carbonate phases were dissolved with 1 M distilled hydrochloric acid. After centrifugation, the supernatant was collected and dried down. A few drops of concentrated distilled nitric acid were added to fully transform the solution matrix before it completely dried down. A small aliquot was used to determine U concentrations in each sample via a Thermo Fisher Scientific iCAP RQ ICP‐MS in the Laboratory for Isotopes and Metals in the Environment (LIME) at Pennsylvania State University. In a clean laboratory, about 195 ng U was further processed for U purification. The IRMM‐3636a ^236^U‐^233^U double spike was added prior to the column chromatography at an average ^236^U:^238^U ratio of 1:50 (*n* = 88). Uranium was purified from other matrix elements by ion exchange chromatography using two passes with UTEVA resin, following Lau et al. (2016). Two drops each of concentrated hydrogen peroxide and nitric acid were added to the eluted U to oxidize any organics contributed from the column. The ^238^U/^235^U ratios were analyzed on a Thermo Fisher Scientific Neptune Plus Multi‐Collector ICP‐MS in the Metal Geochemistry and Geochronology Center (MGGC) at Yale University, with samples diluted to achieve an intensity of 20–30 V for ^238^U. Sample–standard bracketing with CRM‐145 for every three samples was used to calculate δ^238^U_carb_ values. CRM‐129A was analyzed every five samples to determine analytical consistency throughout the run. The sample reproducibility obtained from CRM‐129A is −1.42‰ ± 0.16‰ (average and 2*σ*, *n* = 10). Duplicates of a carbonate in‐house standard (DWP) were processed from powder and produced δ^238^U_carb_ values of −0.23‰ ± 0.13‰ (average and 2*σ*, *n* = 5), in agreement with literature values (Jost et al. 2017; Lau et al. 2016, 2022; Lau, Macdonald, et al. 2017). Measurements of NOD‐A‐1 produced δ^238^U_carb_ values of −0.55‰ ± 0.19‰ (average and 2*σ*, *n* = 5), in agreement with literature values (Goto et al. 2014; Hood et al. 2018; Lau et al. 2022; Tissot and Dauphas 2015; Wang et al. 2018; Weyer et al. 2008). Based on these results, we report the reproducibility to be 0.16‰ (2*σ*).

### 3.2 Carbonate‐Bound Iodine

Following methods from Lu et al. (2010) and Hardisty et al. (2017), ~5 mg of sample powder was cleaned in 1 mL ultrapure water. Following sonication and centrifugation, ~0.8 mL of 3% nitric acid was added to digest carbonate samples. Samples were sonicated until the reaction was complete and then centrifuged. The supernatant was diluted in 0.5% tetramethylammonium hydroxide (TMAH) to achieve a 50 ppm Ca concentration (Hardisty et al. 2017; Lu et al. 2010, 2016). Digestions and analyses were performed on the same day to reduce iodine volatilization (Hardisty et al. 2017). I/(Ca + Mg) ratios were measured with a Thermo Scientific iCAP triple quadrupole (TQ) Inductively Coupled Plasma Mass Spectrometer (ICP‐MS) at Michigan State University. The reproducibility of I/(Ca + Mg) ratios, calculated by repeated measurements of the coral standard JCP‐1, is 4.12 ± 0.17 μmol/mol (average and 1*σ*, *n* = 24), in agreement with previous studies (Chai and Muramatsu 2007; Cook et al. 2022; Lu et al. 2022; Lu et al. 2010). A subset of samples was analyzed in duplicate or triplicate from powder to determine I/(Ca + Mg) reproducibility, and the standard deviations of each sample replica set ranged from 0.003 to 0.14 μmol/mol (with an average of 0.054 μmol/mol, *n* = 20).

### 3.3 Rare Earth Elements and Yttrium (REY) and Elemental Concentrations

The REY concentrations in carbonate rocks were extracted using a selective carbonate sequential dissolution following methods from Tostevin, Shields, et al. (2016). Cao et al. (2020) suggest that filtering the leachate and using weak acetic acid can reduce contamination from non‐carbonate phases. We tested this protocol using a syringe filter (pore size of 0.22 μm), comparing various acid types (acetic acid, hydrochloric acid, and nitric acid), and using a precleaning step with 1 M ammonium acetate (see Data S1 for full details). The Ce/Ce* values were consistent regardless of additional precleaning or filtering steps, or the acid used for digestion (Table S3). Thus, for consistency with previously published Ce/Ce* in upper Ediacaran carbonates, we used 2% distilled nitric acid without a filtration step for REY digestion (Tostevin 2021; Tostevin, Shields, et al. 2016).

For limestones, ~25 mg of powdered samples was sonicated in ultrapure water to remove clay particles and evaporites. After rinsing, 0.333 mL of 2% nitric acid was added to dissolve ~20% CaCO_3_ (by mole). Samples were placed on a shaker table for 20 min during digestion. After centrifugation, the supernatant was decanted, and the residue was rinsed three times in ultrapure water. To dissolve ~40% of targeted carbonate phases, 0.666 mL of 2% nitric acid was added to the residue and allowed to react on a shaker table, and the supernatant was collected and dried on a hotplate. For dolostones, ~50 mg of powdered sample was sonicated in ultrapure water and 0.723 mL of 2% nitric acid was added to dissolve ~20% CaMg (CO_3_)_2_ (by mole). The supernatant was then collected and dried down. All samples were diluted in 2% nitric acid and analyzed via a Thermo Fisher Scientific iCAP RQ ICP‐MS and a Thermo Scientific iCAP 7400 Inductively Coupled Plasma Emission Spectrometry (ICP‐AES) in LIME at Pennsylvania State University. The precision is determined to be within 5% relative standard deviation (RSD) for most trace and all REY elements. A dolomite standard (JDo‐1) was digested using the same procedure, and calculated Ce anomalies (average of 0.29, *n* = 3) and Eu anomalies (average of 1.36, *n* = 3) were used to determine analytical reproducibilities of 0.024 (2*σ*) and 0.016 (2*σ*), respectively.

## 4 Results

### 4.1 Uranium Isotopes (δ^238^U_carb_ )

**FIGURE 3:**
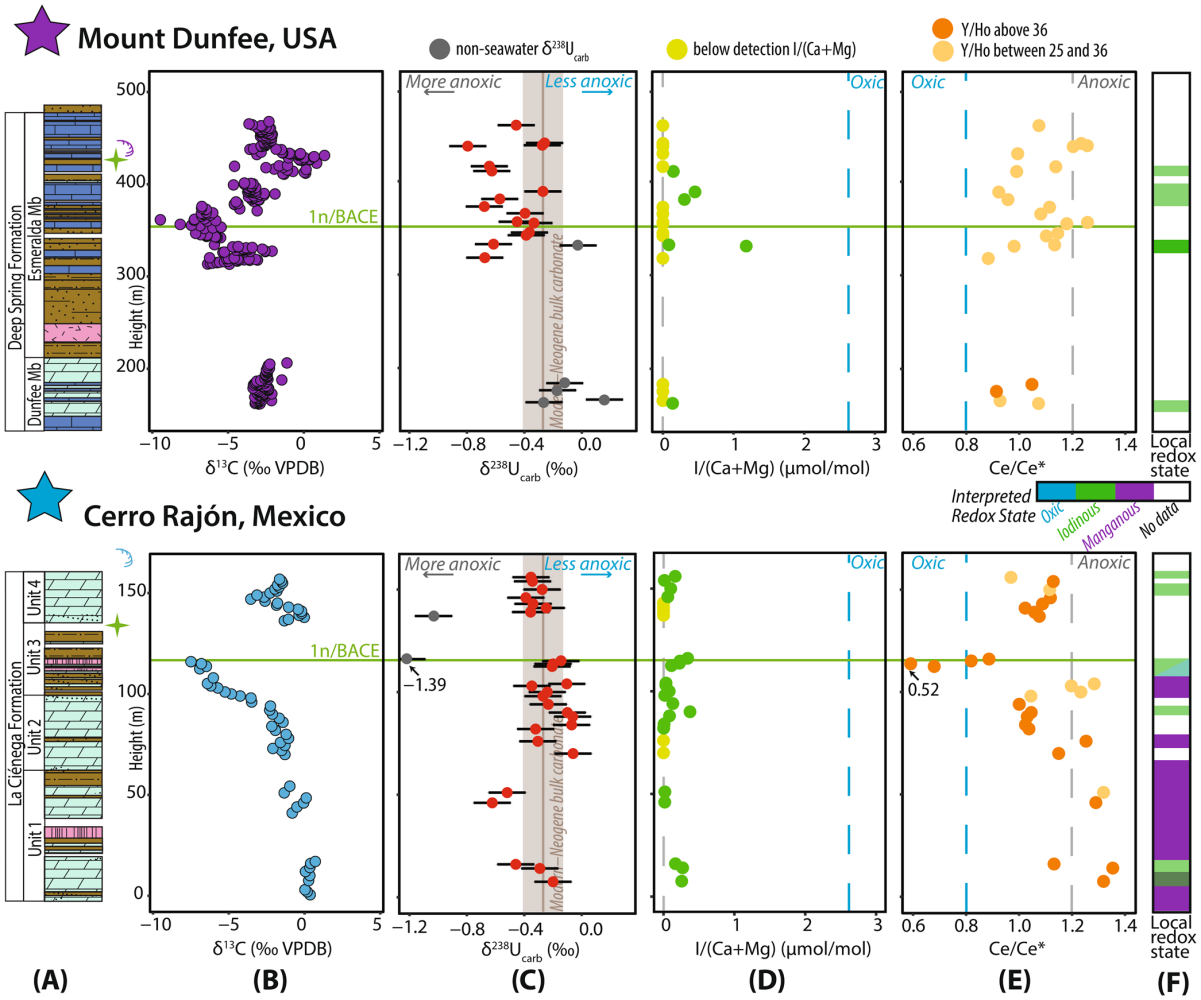
(A) Simplified lithostratigraphy and age constraints for the Deep Spring Formation at Mount Dunfee (upper panel) and the La Ciénega Formation at Cerro Rajón (lower panel) (refer to Figure 1 for detailed stratigraphic columns), (B) δ^13^C chemostratigraphy, (C) uranium isotope (δ^238^U_carb_) data; gray symbols are samples that are interpreted as not representative of seawater (see Section 5.1), (D) carbonate‐bound iodine (I/(Ca + Mg)) data; yellow symbols demarcate samples with ratios below detection, (E) cerium anomaly (Ce/Ce*) data; dark and light orange symbols indicate Y/Ho ratios above and below 36, respectively, and (F) interpretations of seawater redox conditions based on the local redox proxies. Vertical lines: (C) brown = modern–Neogene mean bulk carbonate δ^238^U (−0.27‰ ± 0.14‰ (1*σ*); Chen et al. 2018), (D) blue = modern oxygenated seawater I/(Ca + Mg) ratio (2.6 μmol/mol; Glock et al. 2014; Lu et al. 2016), and (E) defined Ce anomaly thresholds (blue = negative Ce/Ce* < 0.8 and gray = positive Ce/Ce* > 1.2; Tostevin 2021).

In the Mount Dunfee section of the Deep Spring Formation and the Cerro Rajón section of the La Ciénega Formation, we observe variations in δ^238^U_carb_ values of ~0.2‰–0.4‰ but no stratigraphic shifts coincident with the BACE (Figure 3C). In the Deep Spring Formation at Mount Dunfee, δ^238^U_carb_ values range from −0.80‰ to 0.15‰ with an average of −0.40‰ ± 0.19‰ (*n* = 21). The δ^238^U_carb_ values decrease from a mean of −0.10‰ (*n* = 4) in the Dunfee Member to −0.47‰ (*n* = 17) in the overlying Esmeralda Member. The difference in mean δ^238^U_carb_ of the Dunfee and Esmeralda members is statistically significant (*t*‐test; *p*‐value = 0.0174). The δ^238^U_carb_ values reach a minimum of −0.80‰ in the Esmeralda Member. In the La Ciénega Formation at Cerro Rajón, δ^238^U_carb_ values range from −0.62‰ to −0.06‰ with an average of −0.34‰ ± 0.19‰ (*n* = 28). The mean δ^238^U_carb_ is higher in the La Ciénega Formation compared to the Deep Spring Formation, but like at Mount Dunfee, there is no change in δ^238^U_carb_ values coincident with the BACE. In the La Ciénega Formation, mean δ^238^U_carb_ values in Unit 1 increase from −0.42‰ (*n* = 5) to −0.18‰ (*n* = 8) prior to the BACE, and stay relatively invariant with a mean of −0.40‰ (*n* = 15) in units 3 through 4, which contain and overlie the BACE.

### 4.2 Carbonate‐Bound Iodine (I/(Ca + Mg)) Ratios

The I/(Ca + Mg) ratios at all studied sections are predominantly below detection and no samples have values that exceed the threshold of modern oxygenated seawater (2.6 μmol/mol; Figure 3D) (Glock et al. 2014; Lu et al. 2016). The highest individual I/(Ca + Mg) ratio was measured in samples from the Deep Spring Formation, but there are only six samples that have I/(Ca + Mg) ratios above detection. In the Esmeralda Member, the highest I/(Ca + Mg) ratio reaches 1.18 μmol/mol at 331.1 m, but this elevated I/(Ca + Mg) ratio is isolated to one sample. The I/(Ca + Mg) ratios fluctuate between 0.15 and 0.47 μmol/mol through the BACE recovery interval (380.9 to 410.9 m) but then decrease to low and below detection I/(Ca + Mg) ratios up section. Dolostones of the La Ciénega Formation have a higher proportion of samples with I/(Ca + Mg) above detection, but ratios are low. The I/(Ca + Mg) ratios reach a maximum of 0.37 μmol/mol at 90 m of Unit 2 and remain above detection through Unit 3 and the BACE nadir. Four samples in the lower Unit 4 (137 to 143 m) have I/(Ca + Mg) ratios below detection, but the ratios are above detection for the rest of the samples up section.

### 4.3 Cerium Anomalies (Ce/Ce*)

Both studied sections have similar average Ce/Ce* values of ~1 (1.07, *n* = 22 in the Deep Spring Formation and 1.08, *n* = 28 in the La Ciénega Formation), but stratigraphic changes in the Ce anomaly are distinct at each section (Figure 3E). In the Deep Spring Formation, Ce/Ce* values range from 0.88 to 1.26, with four samples having Ce/Ce* values above 1.2. One of these samples with high values is located at the nadir of the BACE (at 356.6 m), and the other samples with high values occur above the BACE (between 437.5 and 440.9 m). In the La Ciénega Formation, Ce/Ce* values are more variable, with a range of 0.52 to 1.35. In Unit 1, the Ce/Ce* values are highest, with most samples > 1.3. The Ce/Ce* values steadily decrease up section and cluster around 1.0 in Unit 2. Unit 3 of this section captures the most variability in Ce/Ce* values. The Ce/Ce* values increase up to 1.3, then suddenly decrease to values below 0.8 and as low as 0.52, between 112.5 m and 115 m, coincident with the nadir of the BACE. Above the BACE, Ce/Ce* values are higher, with values ranging from 0.97 to 1.13.

### 4.4 Rare Earth Elements and Yttrium (REY) and Europium Anomalies (Eu/Eu*)

**FIGURE 4:**
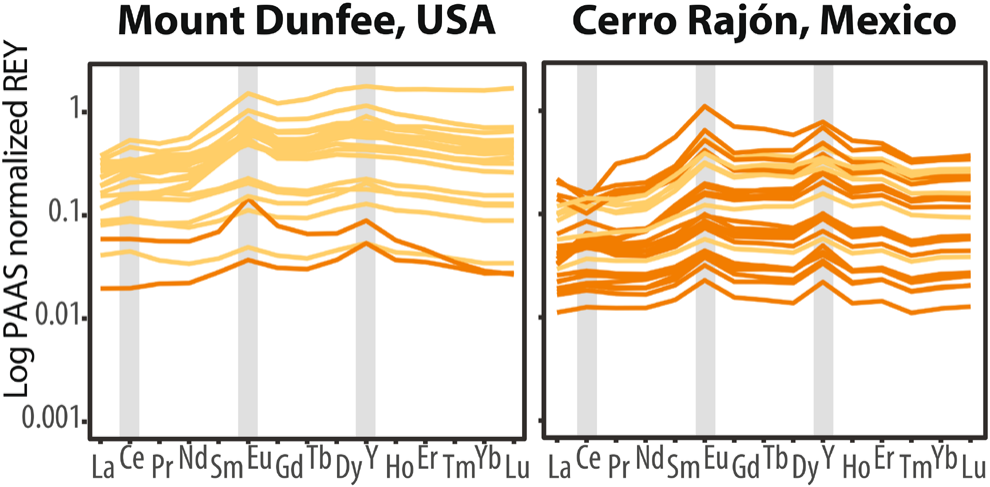
The rare earth elements and yttrium (REY) distribution from the Deep Spring Formation at Mount Dunfee and the La Ciénega Formation at Cerro Rajón, normalized to post‐Archean Australian Shale (PAAS) on a log scale. Ce, Eu, and Y anomalies are highlighted in gray. Dark orange colors indicate Y/Ho > 36; light orange colors indicate Y/Ho between 25 and 36.

Between our sections, REY distributions vary with respect to REY enrichments, Eu anomalies, and Y/Ho ratios (Figure 4; Tables S1 and S2). At Mount Dunfee, REY patterns in the Deep Spring Formation show depleted LREE and enriched HREE, except for six samples that exhibit HREE depletion (i.e., Pr_SN_/Yb_SN_ > 0.8) and MREE enrichment (i.e., Dy_SN_/Sm_SN_ < 1.2) (Figure 4 and Table S1). The average Y/Ho ratio is ~30 (*n* = 22), with only two samples above 36 in the upper Dunfee Member. At Cerro Rajón, REY distributions of samples with Ce/Ce* values < 0.8 display depleted LREE and enriched HREE patterns. Many samples from Units 2 and 4 exhibit minor HREE depletion and MREE enrichment indicated by Pr_SN_/Yb_SN_ and Dy_SN_/Sm_SN_, but Ce/Ce* values are comparable to other samples in these units (Figure 4 and Table S2). The Y/Ho ratios are high and range from ~32 to ~45 (average of ~38, *n* = 28). The Eu/Eu* values generally show small positive or no Eu anomalies at all study sites (average Eu/Eu* 1.50, *n* = 22 in the Deep Spring Formation and 1.53, *n* = 28 in the La Ciénega Formation) and do not covary with Ce anomalies (Figure S1). The Eu/Eu* values reach a maximum of 2.14 in the Deep Spring Formation and 1.98 in the La Ciénega Formation (Figure S1). Higher Eu/Eu* values in these two sections are broadly coincident with the BACE nadir (Figure S1; see Data S1 for Eu anomaly discussion).

### 4.5 Petrographic and Geochemical Evidence for Recrystallization and Diagenesis

To accompany previous lithologic descriptions and interpretations of diagenesis for both sections, nine representative thin sections were selected to better understand the paragenetic features presented in our samples to inform geochemical interpretations (Figure S2 and see Data S1 for full details).

Dolostones of the La Ciénega Formation are predominantly microspar–spar with regions of mm‐scale micrite, suggesting primary marine carbonate preservation (i.e., fabric retentive microcrystalline textures) (Figure S2 7.5‐A). The preponderance of fabric‐retentive microcrystalline dolomite textures—rather than fabric‐destructive textures such as zebra or saddle dolomite—within dolostones of this unit is consistent with early marine diagenesis. Lonsdale et al. (n.d.) suggest a fluid‐buffered regime with seawater‐like fluids at temperatures < 40°C driven by platform brine reflux. Because of the high flux of seawater, these samples likely recorded seawater major element and C and Ca isotope compositions. A sample from the La Ciénega Formation near the BACE nadir also contains dolomite, quartz, and secondary minerals such as clinochlore and goethite, which could indicate burial alteration (Figure S3). However, late‐stage diagenetic features like carbonate‐filled veins and stylolites are limited in these samples and, when present, were avoided during sample powdering.

Limestones of the Deep Spring Formation exhibit variable carbonate crystallization (Figure S2 116 m‐A; 437.5 m‐A) and diagenetic features, such as carbonate veins, stylolites, and subhedral and isolated dolomite crystals (Figure S2 166 m‐B; 332.5 m‐A, B). This section contains diverse mineral grains such as detrital quartz, iron‐oxides, and dolomitic rhombs (Figure S2 380.9 m‐A, B, C). The BACE‐bearing samples from the Esmeralda Member show only a single spar generation, likely resulting from neomorphism (Lonsdale et al., n.d.). The lack of further recrystallization likely supports the retention of seawater geochemical composition of these samples. The recrystallization is not facies specific, and there is no relationship between the degree of fabric retention or late‐stage recrystallization and the major element geochemistry (Lonsdale et al., n.d.).

**FIGURE 5:**
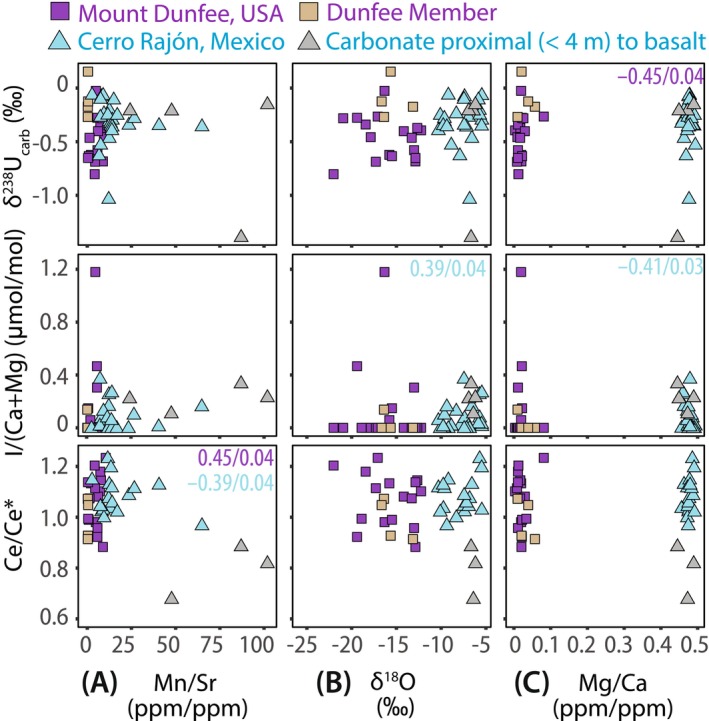
Crossplots of carbonate redox proxies (δ^238^U_carb_, Ce/Ce*, and I/(Ca + Mg)) versus diagenetic indicators: (A) Mn/Sr, (B) δ^18^O, and (C) Mg/Ca. Squares indicate the Deep Spring Formation at Mount Dunfee with tan symbols representing the Dunfee Member. Triangles indicate the La Ciénega Formation at Cerro Rajón and gray symbols indicate samples proximal (< 4 m) to interstratified metabasalt sills. If a correlation is statistically significant (*α* = 0.05), Spearman's rank correlation statistics are shown (purple = Deep Spring Formation and cyan = La Ciénega Formation) as Spearman's *ρ*/*p*‐values (all Spearman's *ρ* and *p*‐value are listed in Table S5).

The top of the Dunfee Member is unevenly dolomitized (e.g., increasing Mg/Ca ratios up section and a karstic dissolution surface at the top of the member) and contains destructive fabrics linked to subaerial exposure and brine reflux (Smith, Nelson, et al. 2016). The late‐state recrystallization with nonplanar dolomite texture is hypothesized to result from crystal regrowth in warm (> 50°C) brine reflux fluids and has largely unaffected the major element geochemistry (Lonsdale et al., n.d.). Although the Dunfee Member samples in our study contain low Mg/Ca values (< 0.1 ppm/ppm; Figure 5C, tan symbols), the interaction with dolomitization could lead to variable geochemical alteration of these samples that controlled the uranium isotope values (see discussion in Section 5.1.3). Aside from the top of the Dunfee Member, we do not find textural evidence for recrystallization as a controlling factor on the redox proxy results within the sample set.

## 5 Discussion

### 5.1 Distinguishing Seawater Signals in Carbonate Geochemical Records

Before interpreting geochemical records as seawater paleoredox indicators, it is important to assess overprinting by non‐seawater fluids. We use our integrated data to determine the potential influence of hydrothermal fluids, meteoric diagenesis, and dolomitization on our paleoredox proxy records. Because each alteration process affects geochemical proxies differently, they must be evaluated independently to distinguish seawater conditions from diagenetic overprinting.

#### 5.1.1 Constraints on Hydrothermal Influence on Paleoredox Proxies

Our geochemical data suggest localized hydrothermal influence within limited stratigraphic intervals. A Mn/Sr threshold < 10 has been previously used to identify seawater δ^13^C signals in Proterozoic carbonates (e.g., Azmy et al. 2011; Bartley et al. 1998; Gilleaudeau et al. 2018; Kaufman and Knoll 1995). All Deep Spring samples exhibit Mn/Sr < 10 (Figure 5A). In contrast, La Ciénega dolostones generally show higher ratios that are < 15, which may reflect diagenesis under reducing conditions or the presence of hydrothermal fluids enriched in Mn. Eight samples from Units 3 and 4 have Mn/Sr ratios > 20, with two reaching ~102 and ~87 at 115 m and 116 m, respectively (Figure 5A and Table S2). Two of these samples with high Mn/Sr ratios (at 112.5–116 m) are located proximally to interstratified metabasalt sills (Barrón‐Díaz et al. 2019; Hodgin et al. 2021; Tapia‐Trinidad et al. 2022), supporting localized Mn addition from hydrothermal fluids (Moffett and German 2020; Resing et al. 2015; von Damm 1990).

Despite elevated Mn/Sr ratios in the La Ciénega Formation that suggest hydrothermal input, we do not find evidence that local redox proxies (I/(Ca + Mg) and Ce/Ce*) were impacted. Although hydrothermal fluids may increase total iodine from organic‐rich sediments, these reducing fluids release iodide, which is not incorporated into carbonates (Campbell and Edmond 1989; Magenheim and Gieskes 1992; You et al. 1994). Under strongly reducing conditions where iodate is reduced to iodide, I/(Ca + Mg) ratios would be expected to fall below detection. However, our I/(Ca + Mg) records adjacent to basalt sills remain above detection (Figure 3D, green symbols), suggesting the presence of iodate and limited overprinting by hydrothermal alteration. Hydrothermal fluids would be expected to contain high Fe, Mn, and positive Eu and Ce anomalies (Bau 1991; Möller 2002), but samples with high Mn/Sr (between 112.5 and 116 m) appear to retain seawater‐like REY patterns, with Y/Ho > 36 and relatively low Fe (Figure 4 and Table S2). The small positive Eu/Eu* values in our samples (~1.7–2) align with other Precambrian carbonate samples that range from 0.9 to 2.4 (e.g., Chen et al. 2014; Hohl et al. 2017; Kamber and Webb 2001; Ling et al. 2013). Contamination from Fe‐Mn (oxyhydr)oxides can also elevate Mn and mask seawater Ce anomalies (Cao et al. 2020; Zhang and Shields 2023), but this contribution is minimized by our sequential acid digestion. No patterns indicative of oxide influence and remobilization—bell‐shaped index, MREE enrichment, and correlation of Ce anomalies to Fe and Mn concentrations—are observed (Figure S4 upper panel, Table S2, see Data S1 for full details). Therefore, we do not find evidence for hydrothermal alteration nor Mn oxide contamination that caused the negative Ce anomalies (i.e., oxidizing conditions) observed in these samples (Figure 3E). The extent of metabasalt sill alteration appears to have affected selective elemental concentrations but did not completely overprint the local redox signals. We interpret I/(Ca + Mg) and Ce anomaly variability in the La Ciénega Formation to record shallow seawater redox conditions. In Section 5.4, we discuss the potential for hydrothermal input (from slightly elevated Eu/Eu*) to correspond to local oxygenation (from negative Ce anomalies) and the BACE.

We also find no evidence that hydrothermal fluids or Mn oxides influenced δ^238^U_carb_ values (Figure 5A). Ferromanganese (Fe–Mn) sediments exhibit U isotope fractionation of ~−0.24‰ (Brennecka et al. 2011; Goto et al. 2014) and produce an inverse correlation between Mn concentrations and δ^238^U_carb_, which is not observed in our data set (Figure S5). We note two samples from Cerro Rajón with anomalously low δ^238^U_carb_ of −1.39‰ and −1.03‰ at 116 m and 137 m respectively (Figure 3C, gray symbols) that reflect unusual isotope fractionation. Although high‐temperature basalt alteration is not expected to fractionate U isotopes, carbonate veins within altered basalts show heterogeneous δ^238^U_carb_ composition (ranging from −0.63‰ to +0.11‰) likely due to induced selective reduction of U (VI) during hydrothermal alteration (Andersen et al. 2014; Noordmann et al. 2016). These low δ^238^U_carb_ values also fall below the range of potential seawater δ^238^U based on mass balance calculations using reasonable isotope fractionation factors for anoxic sediments (Lau, Macdonald, et al. 2017), suggesting localized U isotope fractionation during hydrothermal alteration rather than global redox change of primary seawater. Therefore, we do not interpret these two samples to record seawater δ^238^U trends.

#### 5.1.2 Constraints on Meteoric Influence on Paleoredox Proxies

Carbonate oxygen isotopes are a useful tool to evaluate early meteoric diagenesis, typically characterized by low δ^18^O values (< −10‰) (e.g., Allan and Matthews 1982; Brand and Veizer 1980; Derry et al. 1994; Kaufman and Knoll 1995; Veizer et al. 1999). However, low δ^18^O values (< −15‰ to −20‰) from lower Cambrian carbonate strata do not always correlate with δ^13^C, suggesting δ^18^O alone may not be a reliable indicator of altered carbonates (e.g., Chang et al. 2017; Ishikawa et al. 2008; Li et al. 2013; Wotte et al. 2007). Warm pore fluids can also lower δ^18^O without affecting δ^13^C during burial diagenesis. In this study, low δ^18^O values (< −12‰) in the Deep Spring Formation may indicate meteoric and/or burial diagenesis, but there is no correlation between δ^18^O values and δ^238^U_carb_ or Ce/Ce* values (Figure 5B), implying minimal impact on paleoredox proxies. The correlation between δ^18^O and I/(Ca + Mg) data (Spearman's *ρ* = 0.39, *p*‐value = 0.04) is unlikely to indicate significant meteoric alteration, because δ^18^O values in the La Ciénega Formation are greater than −10‰.

In addition to meteoric diagenesis, freshwater fluids can extend into marine environments and influence REY distributions and the Ce anomaly (e.g., Novais et al. 2024; Nozaki et al. 1997; Tepe and Bau 2016). Seawater‐like REY patterns are characterized by HREE enrichment relative to LREE, low total rare earth element concentrations (ΣREE), small‐to‐absent positive Eu anomalies, and Y/Ho > 36 (e.g., Alibo and Nozaki 1999; Cao et al. 2020; de Baar et al. 1985; Elderfield 1988; Liu et al. 2019; Shields and Stille 2001; Tostevin, Shields, et al. 2016; Zhang and Shields 2023). The majority of La Ciénega carbonates display these features (Figure 4), supporting marine Ce anomaly preservation in these samples. In contrast, the Deep Spring samples have high ΣREE (~50 ppm, *n* = 22) and Y/Ho < 36 (light orange symbols in Figure 4E and Figure S6, and see Data S1 for full details). Detrital clays and oxides can produce non‐marine REY signatures, such as a flat REY pattern and Y/Ho of ≤ 25 (Frimmel 2009; Nothdurft et al. 2004; Tostevin, Shields, et al. 2016), but our sequential acid leaching method minimized contamination and yielded reproducible Ce anomaly and REY data (Figure S7 and Table S3). Therefore, we attribute the REY patterns and low Y/Ho of the Deep Spring Formation to reflect local freshwater mixing and continental REY input (Bau et al. 1996; Kamber et al. 2005; Lawrence and Kamber 2006; Novais et al. 2024; Nozaki et al. 1997). Therefore, we conservatively do not interpret the Ce anomaly data at Mount Dunfee in our reconstruction of marine redox changes (see Data S1 for full details). Freshwater input likely did not affect δ^238^U_carb_ records, as seawater U is conservative and well‐mixed in the ocean (Dunk et al. 2002; Tissot and Dauphas 2015). While riverine fluids have larger organic iodine and iodide pools than seawater, marine iodine speciation is conservative with salinity (Jones et al. 2024; Luther et al. 1991; Moran et al. 2002; Ullman et al. 1988; Yoshida et al. 2007), and thus our I/(Ca + Mg) ratios reflect seawater iodate availability.

#### 5.1.3 Constraints on Proxy Preservation During Dolomitization and Marine Diagenesis

Dolomitization requires significant fluid fluxes (Land 1985; Warren 2000). If these fluids are seawater, early marine dolomitization may preserve seawater paleoredox signals. Mg/Ca ratios are used to indicate the extent of dolomitization and are not systematically correlated with Ce anomaly at either study site (Figure 5C and Figure S8), suggesting minimal alteration during dolomitization. An inverse correlation is observed between Mg/Ca and I/(Ca + Mg) ratios at Cerro Rajón (Spearman's *ρ* = −0.41, *p*‐value = 0.03), supporting previous findings that dolomitization may reduce iodate in carbonates (Hardisty et al. 2017). However, changes in I/(Ca + Mg) are not observed with stratigraphic variations in dolomite (Figure 3D, see Section 4.5), and therefore the stratigraphic patterns in I/(Ca + Mg) are not a function of only dolomitization.

**FIGURE 6:**
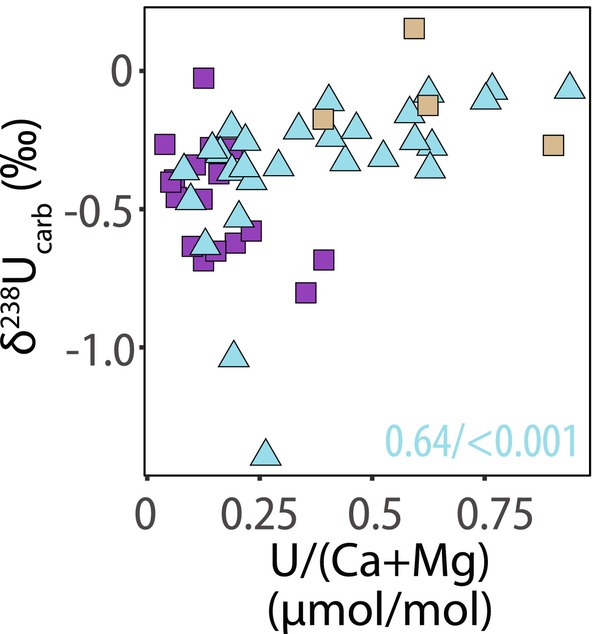
Crossplot of δ^238^U_carb_ versus U/(Ca + Mg) ratios. Purple squares indicate the Deep Spring Formation at Mount Dunfee with tan symbols representing the Dunfee Member. Cyan triangles indicate the La Ciénega Formation at Cerro Rajón. Spearman's rank correlation statistics are only significant (*α* = 0.05) for the La Ciénega Formation and are shown as Spearman's *ρ*/*p*‐value (all Spearman's *ρ* and *p*‐value are listed in Table S5).

Diagenetic porewater uranium reduction can alter uranium isotope values (e.g., Chen et al. 2018; Tissot et al. 2018). We hypothesize that this process could have been associated with dolomitization in the upper Dunfee Member and the La Ciénega Formation, leading to elevated δ^238^U_carb_ compared to the coeval Esmeralda Member of the Deep Spring Formation (See Figure 6). The uranium isotope fractionation associated with dolomitization is not known (Romaniello et al. 2013). However, if dolomitizing fluids are only slightly evolved from seawater, seawater δ^238^U values may be preserved in carbonates. Previous studies have shown that dolostones can preserve δ^238^U_carb_ values comparable to limestones (e.g., Chen et al. 2018; Gilleaudeau et al. 2019; Herrmann et al. 2018). However, dolomitization can occur under variable diagenetic conditions, which could result in distinct impacts on the δ^238^U_carb_ values of a given dolomite sample (cf. Herrmann et al. (2018) in Figure S9). If dolomitization occurred with reducing porewaters—even if derived from seawater—U reduction could occur and result in large, positive diagenetic offsets. We hypothesize that early dolomitization with reducing porewaters could have been more pronounced in the Precambrian if absent or limited bioturbation led to enhanced organic matter preservation and anaerobic remineralization in sediments, promoting uranium porewater reduction. This hypothesis could be tested in future studies at other sites.

The hypothesis that dolomitization of La Ciénega and upper Dunfee Member samples was accompanied by enhanced uranium reduction in porewaters is supported by a hyperbolic relationship between U/(Ca + Mg) and δ^238^U_carb_ (Figure 6). Elevated U isotope values and increased U/Ca ratios are expected from U (VI) reduction and U (IV) accumulation during early marine diagenesis, a process that could result in higher δ^238^U_carb_ values because of a larger diagenetic isotope offset between carbonate and seawater. To illustrate, the average isotope offset between seawater and carbonates observed in modern to Neogene carbonate sediments is +0.25‰, but with a large variance that suggests porewater U reduction can increase δ^238^U_carb_ by up to 0.5‰ during early diagenesis (e.g., Chen et al. 2018; Romaniello et al. 2013; Tissot et al. 2018). Similarly, carbonate sediments precipitated under euxinic conditions in the meromictic Fayetteville Green Lake display a large isotope offset of +0.4‰ attributed to the presence of uranium reduction (Chen, Romaniello, et al. 2021). This effect is also observed in carbonate rock records. A range in δ^238^U_carb_ of > 0.3‰ in carbonates that span the Permian–Triassic boundary represents diagenetic offsets that differ by diagenetic regime unique to each section (e.g., Brennecka et al. 2011; Elrick et al. 2017; Lau et al. 2016; Zhang, Romaniello, et al. 2018; Zhang, Shen, et al. 2020). On the other hand, these examples all support our hypothesis that dolomitization was linked to U reduction during early marine diagenesis, resulting in δ^238^U_carb_ values in carbonates that are ~0.2‰ higher in the La Ciénega Formation compared to coeval δ^238^U_carb_ values in the Esmeralda Member of the Deep Spring Formation (Figure 3C).

The style of dolomitization also differs between the two sections of this study, resulting in varying redox interpretations of carbonate associated uranium isotopes. The dolomitization in the La Ciénega Formation preserves microcrystalline fabrics and is interpreted to record early marine diagenetic conditions (see Section 4.5). We used calcium isotope data (after Lonsdale (2025); Lonsdale et al. n.d.) to assess diagenetic overprinting and the effect of recrystallization on our paleoredox proxies based on diagenetic model predictions (Lau and Hardisty 2022). The crossplot of δ^44/40^Ca against δ^238^U_carb_ values from the La Ciénega Formation does not exhibit any clear Ca and U isotope trends following the modeled diagenetic trajectories (Figure S10A). Thus, we argue that seawater‐buffered diagenesis characterizes the La Ciénega samples, supporting these dolostones as an archive of seawater δ^238^U trends. In other words, the δ^238^U_carb_ variations in these samples are interpreted to reliably track first‐order changes in seawater δ^238^U over time, albeit with a larger diagenetic offset than in coeval limestones. In contrast, upper Dunfee Member limestones (low Mg/Ca < 0.1 ppm/ppm; Figure 5C, tan symbols) have been influenced by diagenesis, such that they cannot be used to constrain changes in seawater δ^238^U. The crossplot of δ^44/40^Ca versus δ^238^U_carb_ in the Deep Spring Formation shows a relationship unique to our dataset that indicates diagenetic alteration drove the changes in the uranium isotope values of upper Dunfee Member samples (Figure S10A). The four samples from the upper Dunfee Member show increasing δ^44/40^Ca (−1.6‰ to −1.2‰) with increasing δ^238^U_carb_ values that are much higher than continental inputs, up to 0.2‰. This trajectory matches the model prediction for diagenetic recrystallization with reducing seawater (see red dashed line in Figure S10A based on Lau and Hardisty (2022) prediction). Therefore, the changes in δ^238^U_carb_ values of these samples do not record a change in seawater but instead diagenetic alteration associated with increasing recrystallization. Thus, we exclude the Dunfee Member samples (gray symbols in Figure 3C) from the δ^238^U_carb_ compilation and further interpretation. In addition, we note that no similar trajectory is observed for the other uranium isotopes and local redox proxies of Ce/Ce* and I/(Ca + Mg) (Figure S10B,C) from the Deep Spring Formation, and therefore we find evidence that this alteration was limited to a subset of uranium isotope data (see Data S1 for full details).

In summary, the δ^238^U_carb_ values in the La Ciénega Formation may reflect secular seawater changes, with a consistent, larger positive isotopic offset due to greater uranium reduction during early dolomitization (Figure 3C, red symbols). In contrast, elevated δ^238^U_carb_ values in the Dunfee Member likely reflect variable diagenetic overprinting (Figure 3C, gray symbols), distinct from the overlying Esmeralda Member. Accounting for these diagenetic impacts, first‐order δ^238^U_carb_ observations at both sites show no δ^238^U_carb_ trends across the BACE, suggesting no associated global redox shifts across the Ediacaran–Cambrian boundary.

### 5.2 Interpretations of Shallow Marine Local Redox Conditions from Ce Anomaly and Carbonate‐Bound Iodine Proxies

Samples with I/(Ca + Mg) ratios above detection provide strong evidence that iodate was present in seawater, which requires the presence of dissolved oxygen (Figure 3D, green symbols). Ratios of iodine‐to‐calcium‐magnesium in carbonate rocks reflect iodate abundance, and thus low I/(Ca + Mg) ratios can indicate reducing water column conditions with iodate reduction. Overall, I/(Ca + Mg) ratios in our study—reaching maximum values of 1.18 and 0.37 μmol/mol in the Deep Spring and La Ciénega formations, respectively—are well below the threshold of 2.6 μmol/mol that characterizes modern oxic seawater (Glock et al. 2014; Lu et al. 2016) (Figure 3D). Therefore, our I/(Ca + Mg) records suggest a regionally redox‐stratified ocean and/or locally reducing surface oceans in southwestern Laurentia through the terminal Ediacaran and early Cambrian. Locally, iodinous conditions—i.e., seawater with dissolved O_2_ levels low enough to host active iodate reduction—would lead to low iodate concentrations, as suggested by the low I/(Ca + Mg) range observed at all sections. Low, but present, iodate could also reflect a regionally redox‐stratified ocean, even if surface waters were oxygenated (Hardisty et al. 2021). This is possible because the kinetics of oxidation of iodide to iodate are slow at half‐lives of years to decades (e.g., Cheng et al. 2024; Fentzke et al. 2025; Hardisty et al. 2020; Moriyasu et al. 2020; Schnur et al. 2024), whereas the reduction of iodate can occur rapidly on the timescale of hours or less (Farrenkopf et al. 1997; Hardisty et al. 2021). Thus, the regional accumulation of iodate also depends on the relative residence time of waters in shallow oxygenated settings hosting slow iodide oxidation versus in deeper anoxic settings with faster iodate reduction. For example, seasonal vertical mixing or circulation patterns could maintain low iodate in oxic surface waters through intermittent introduction of high iodide/low iodate waters from more reducing settings via upwelling.

Similar to carbonate‐bound iodine, the Ce anomaly can identify changes in local shallow marine redox conditions. Lower Ce concentrations relative to its neighboring elements can indicate dissolved oxygen levels sufficiently high for Mn‐ and Fe‐ oxides to form. The sorption of Ce to Mn‐ and Fe‐oxides results in a negative Ce anomaly (Ce/Ce* < 0.8) that can be recorded in carbonates. Here we focus interpretation on the Ce anomaly data from the La Ciénega Formation at Cerro Rajón (see Section 5.1.2). Both negative and positive Ce anomalies are observed in the La Ciénega Formation, suggesting localized redox variability above and below the manganous zone along the southwestern Laurentian margin at the Ediacaran–Cambrian boundary. The positive Ce anomalies in Units 1 and 2 of the La Ciénega Formation indicate that the shallow ocean was locally anoxic (manganous or more reduced) leading up to the BACE interval (Figure 3E). At the BACE nadir, samples with negative Ce anomalies indicate a shift to locally oxic conditions above the manganous zone. These samples in Unit 3 also contain low but above detection I/(Ca + Mg) ratios, suggesting the presence of sufficient dissolved oxygen to maintain low levels of iodate (Figure S11). This oxic interval—with enough dissolved oxygen to be above the Mn reduction threshold but potentially at levels below which a modern‐like iodate pool could be established—was limited temporally (i.e., observed only in ~2.5 m of stratigraphy). Then Ce anomalies return to higher Ce/Ce* values in Unit 4. If these proxy records track paleoenvironmental changes, a localized and transient oxygenation associated with the nadir of the BACE is the most parsimonious explanation. Overall, this suggests changes in oxygen levels were limited, transient, and relatively localized across the Ediacaran–Cambrian boundary.

Based on the local proxy records, the shallow oceans along the southwestern Laurentian margin were locally redox stratified, with surface waters that were iodinous to manganous. Intervals with higher I/(Ca + Mg) ratios—which can be challenging to confidently interpret as higher seawater iodate levels given this proxy is particularly susceptible to diagenesis—are recorded in a limited number of samples. Recorded in the La Ciénega Formation, shallow marine environments exhibited variable local redox conditions leading up to the early Cambrian, with evidence of predominantly anoxic (manganous) conditions that locally transitioned to more oxidized conditions coincident with the BACE nadir. In sum, our study sites do not record any sustained or widespread increases in oxygenation across the BACE interval to a degree comparable to the modern ocean.

**FIGURE 7:**
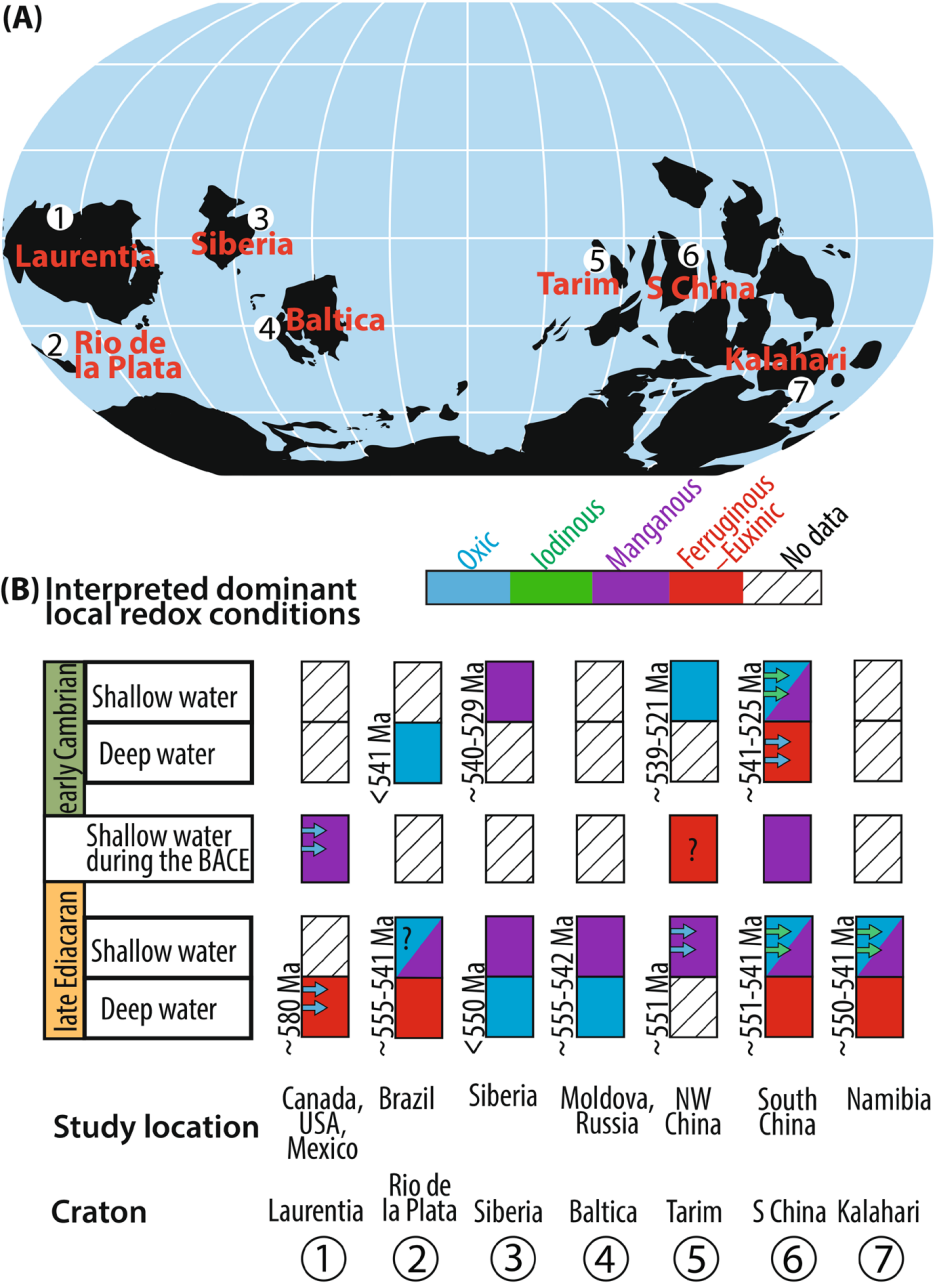
(A) Spatial distribution of local redox proxy records on a 540 Ma paleogeographic reconstruction (modified from Bowyer et al. 2024; Merdith et al. 2021) and (B) interpretations of dominant redox conditions in shallow‐ and deep‐water settings along different paleocontinental margins during the BACE and the Ediacaran–Cambrian transition. Colors indicate redox potential (see also Figure 2). Arrows indicate transient redox fluctuations. Interpretations are from carbonate and shale records of Fe speciation, Ce/Ce*, and I/(Ca + Mg) proxies (see description in text and in references listed in Table S4). The interpreted age range is shown for each location.

Our data, which constrain the redox state associated with the BACE interval in southwestern Laurentia, corroborate the overall picture of heterogeneous local redox conditions in Ediacaran–Cambrian shallow oceans (e.g., Bowyer et al. 2017; Cole, Mills, et al. 2020; Wood et al. 2015, 2019). Evidence from Ce anomaly and Fe speciation data in the Nama Group in Namibia suggests an overall redox‐stratified water column, with anoxic deep waters and oxygenated surface waters (Tostevin, Wood, et al. 2016) (Figure 7, Kalahari craton). Oxygenation of shallow environments is supported by I/(Ca + Mg) ratios as high as 6.20 μmol/mol in the Kuibis Subgroup of the lower Nama Group (Uahengo et al. 2020), skeletal animal fossils, and burrows (Tostevin, Wood, et al. 2016; Wood et al. 2015). Meanwhile, local redox conditions elsewhere, recorded by carbonate‐based Ce anomaly from Oman, Siberia, and the East European Platform, indicate largely anoxic seawaters during the Ediacaran–Cambrian transition (Cherry et al. 2022; Francovschi et al. 2020; Schröder and Grotzinger 2007) (Figure 7, Siberia craton). A combination of Fe speciation and Ce anomaly data from the Corumbá Group in Brazil suggests surface waters were redox stratified in western Gondwana (Fernandes et al. 2024; Spangenberg et al. 2014), despite a coeval shift in bottom waters from anoxic to oxic conditions as sea level rose in the early Cambrian (Caxito et al. 2024) (Figure 7, Rio de la Plata craton). Dynamic redox conditions across basin, slope, and outer shelf environments are identified in South China (Ding et al. 2022; Wei et al. 2024). In the Yangtze Block, South China, Ce anomaly records in upper Ediacaran–lower Cambrian strata have identified frequent redox fluctuations of shallow waters and show systematically decreasing Ce/Ce* values from the late Ediacaran into the early Cambrian, suggesting a broad increase in shallow‐water oxygenation (Ling et al. 2013; Wei et al. 2018, 2024; Wei, Planavsky, et al. 2020; Wei, Frei, et al. 2020) (Figure 7, South China craton). In addition, redox conditions of the surface ocean were spatially heterogeneous, as Ce/Ce* and I/(Ca + Mg) proxies analyzed from two coeval sections in South China exhibit distinct records of redox change (Ding et al. 2022; Ling et al. 2013). In the Tarim basin in northwest China, Ce anomaly data from upper Ediacaran strata record low oxygen conditions, with seawater becoming gradually oxic in the early Cambrian, coincident with local faunal diversification (Liyuan et al. 2021) (Figure 7, Tarim craton).

Characterization of local redox changes associated with the BACE is more limited (Figure 7 and Table S4). Previous Ce/Ce* studies from the Ediacaran–Cambrian carbonate strata in South China have proposed that, locally, the shallow ocean transiently shifted to more anoxic conditions coincident with the prominent negative excursion, interpreted as the BACE (Ling et al. 2013; Yang et al. 2023). These results differ from our combined local redox results (Ce anomaly and I/(Ca + Mg) ratios) from southwestern Laurentia, which either show a brief oxygenation event (La Ciénega Formation) or no clear redox changes (Deep Spring Formation). Therefore, redox shifts are not globally consistent across the BACE interval, supporting our conclusion that local redox conditions in shallow oceans were spatially heterogeneous. Sites in the Tarim Basin, northwest China, and in the Olenek Uplift, Siberia may partially record the BACE (Cherry et al. 2022; Liyuan et al. 2021), but the absence of biostratigraphic constraints as well as the potential for significant hiatuses makes correlations challenging (e.g., Bowyer, Zhuravlev, et al. 2023; Topper et al. 2022; Yang et al. 2016; Zhu et al. 2017; Zhuravlev et al. 2012). This synthesis of local redox proxies from multiple paleo‐continents supports the potential for shallow oceans to have experienced transient and localized redox changes with the BACE (Figure 7), with brief oxygenation identified in one site in Laurentia associated with the BACE nadir.

### 5.3 Interpretations of Global Redox Conditions During the BACE

**FIGURE 8:**
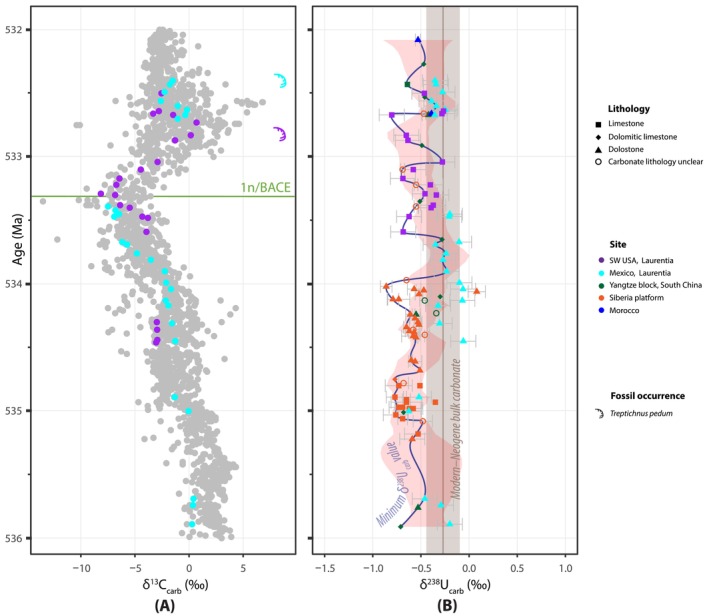
(A) The carbonate carbon isotope records during the BACE (ca. 536–532 Ma), based on “model K” from Bowyer et al. (2022); Bowyer, Uahengo, et al. (2023). The green line labeled “1n” corresponds to the BACE nadir after Nelson et al. (2023). The fossil symbol, colored by study locality, indicates the lowest fossil occurrence of *T. pedum* (Hodgin et al. 2021; Nelson et al. 2023). (B) The carbonate‐associated uranium isotope record, modified from Bowyer et al. (2024). Symbols for uranium isotope data indicate lithology and colors indicate site. The red curve is a LOESS regression of the compilation (samples considered non‐representative of seawater, marked with gray symbols in Figure 3C, are excluded). Data are shown from southwestern Laurentia (this study), Yangtze Block, South China (Wei et al. 2018; Zhang, Xiao, et al. 2018), Siberian platform (Cherry et al. 2022; Dahl et al. 2019), and Morrocco (Dahl et al. 2019). Vertical brown line = modern–Neogene mean bulk carbonate δ^238^U (−0.27‰ ± 0.14‰ (1*σ*); Chen et al. 2018). The solid navy curve denotes the minimum carbonate δ^238^U from this compilation (following Dahl et al. 2019). Based on the observation that early marine diagenesis results in only positive shifts in carbonate δ^238^U, we use the lowest δ^238^U_carb_ data points in the compilation to mark the lowest δ^238^U_carb_ value, with the higher co‐eval δ^238^U_carb_ values interpreted to have been impacted to a greater extent by diagenesis. At most intervals, this minimum value is below the Modern–Neogene bulk carbonate δ^238^U value, indicating that the ocean must have been more anoxic. This curve does not represent changes in seawater δ^238^U over time.

The BACE has been argued to represent a global carbon cycle perturbation in the lead‐up to the Ediacaran–Cambrian boundary (Amthor et al. 2003; Darroch et al. 2018; Smith, Nelson, et al. 2016). However, changes in ocean oxygenation in relation to the BACE are not well‐characterized. Bowyer et al. (2024) proposed that the falling limb of the BACE is coincident with global oxygenation, based on a positive carbonate uranium isotope excursion identified in compiled δ^238^U_carb_ records. The δ^238^U_carb_ compilation in Bowyer et al. (2024) (ca. 541–530 Ma) is based on studies from the uppermost Ediacaran and lowermost Cambrian strata in the Yangtze Block of South China (Wei et al. 2018; Zhang, Xiao, et al. 2018), Siberia Platform (Cherry et al. 2022; Dahl et al. 2019), and Morocco (Dahl et al. 2019). None of these mentioned sites confidently preserve a complete record of the BACE (Figure 8) and therefore cannot be used directly to determine if a global redox change accompanied the negative carbon isotope excursion. For instance, carbonates from the upper Ediacaran Turkut Formation of the Olenek Uplift in Siberia have low δ^238^U_carb_ values of −0.57‰ ± 0.08‰, which are interpreted as widespread marine anoxia (Cherry et al. 2022). This study does not exhibit any δ^238^U_carb_ stratigraphic shifts during the late Ediacaran into the early Cambrian, and the BACE is not preserved in this section due to regional truncation (Bowyer, Zhuravlev, et al. 2023; Cherry et al. 2022). In South China, a negative δ^13^C excursion in the lowermost Cambrian strata near the Ediacaran–Cambrian boundary is coincident with a positive δ^238^U_carb_ excursion (Wei et al. 2018; Zhang, Xiao, et al. 2018). This negative carbon isotope excursion has been proposed to be the BACE (e.g., Topper et al. 2022; Yang et al. 2016; Zhu et al. 2017), but this identification is complicated due to the condensed stratigraphy, likely representing extensive depositional hiatuses, and the absence of *T. pedum* that necessitates other regional biostratigraphic markers (Topper et al. 2022; Yang et al. 2023). The record of carbonates δ^238^U from the Oued Sdas section, Anti‐Atlas Mountains in Morocco begins above the BACE and is included in the compilation for completeness (Dahl et al. 2019) but cannot be used to constrain redox changes directly associated with the carbon isotope excursion. Further, the δ^238^U_carb_ records are sampled at relatively low resolution in association with the potential BACE.

Our updated δ^238^U_carb_ compilation does not identify a clear shift in global redox conditions from ca. 536–532 Ma associated with the onset, nadir, or recovery of the BACE (see red LOESS curve in Figure 8; c.f. Bowyer et al. 2024). The large variability in the uranium isotope records across this interval may reflect differences among stratigraphic sections driven by differences in mineralogy, facies, lithology, and/or diagenesis, rather than by coherent global redox change. As described in Section 5.1.3, we hypothesize that dolomitization under the influence of uranium porewater reduction in the La Ciénega Formation could result in a positive uranium isotope diagenetic offset, which could partially explain the range observed in the carbonate‐associated uranium isotope compilation. The higher δ^238^U_carb_ values across the Ediacaran–Cambrian transition are generally associated with dolostones from the La Ciénega Formation, which are different from syndepositional δ^238^U_carb_ records (Figure 8; Siberia platform and Yangtze block carbonates). To infer seawater δ^238^U values, we follow previous studies that assume that the average diagenetic isotope offset is always positive (e.g., Chen et al. 2018; Romaniello et al. 2013; Tissot et al. 2018), which implies that the lowest δ^238^U_carb_ value from multiple contemporaneous stratigraphic sections can constrain the minimum carbonate δ^238^U value (e.g., Clarkson et al. 2020; Dahl et al. 2019; see solid navy curve in Figure 8). This minimum δ^238^U_carb_ value during the Ediacaran–Cambrian boundary is below the modern–Neogene bulk carbonate δ^238^U value of −0.27‰ (Chen et al. 2018) at most intervals, with the exception of a brief period where the record only is defined by the dolomitized samples from the La Ciénega Formation. Because these samples are interpreted to have experienced a larger diagenetic offset, as a whole the constraints on the minimum δ^238^U_carb_ value suggest a more globally anoxic ocean (i.e., relative to above the Fe redox potential zone) compared to today.

### 5.4 Redox Conditions, Carbon Perturbations, and Metazoan Evolution

We explore two potential hypotheses to explain a global negative δ^13^C excursion without widespread changes in redox conditions. In the canonical interpretation of carbon isotopes (Broecker 1982; Kump 1991; Kump and Arthur 1999), a negative carbon isotope excursion reflects a smaller fraction of organic carbon burial relative to total carbon burial. In this model, a lower organic carbon burial flux could reflect a more oxygenated ocean, which would more readily oxidize organic carbon and reduce burial rates. Alternatively, on long time scales, lower organic carbon burial would lead to a more anoxic ocean because the long‐term removal of reductants such as organic carbon is a source of oxygen. This canonical framework implies a close coupling between negative carbon isotope excursions and redox changes. Instead, we propose that local processes could explain the decoupling of the carbon isotope record and redox conditions. Two hypotheses—which are non‐mutually exclusive—include local marine processes and volcanism.

First, the BACE could arise from the upwelling of deep waters with a large dissolved organic carbon pool, which formed in a largely anoxic ocean where aerobic respiration was absent (Liyuan et al. 2021; Rothman et al. 2003; Yang et al. 2020). In shallow, more oxygenated waters, this organic carbon pool would become oxidized, resulting in a negative carbon isotope excursion. Redox proxies indicate that the BACE was associated with transient and localized oxygenation—such as the negative Ce anomalies observed at Cerro Rajón and a shift from anaerobic to aerobic nitrogen recycling in Central Iberia, Spain (Zhang et al. 2024). Spatially heterogeneous distribution of dissolved oxygen in the shallow ocean could reflect low atmospheric *p*O_2_ levels of 1%–10% PAL (Hardisty and Lau 2024; Reinhard et al. 2016). Variable oxygenation of the shallow ocean may have occurred in redox zones above the Fe reduction redox potential (Figure 2; i.e., oxic–iodinous–manganous zones), which would not have impacted the δ^238^U_carb_ proxy and interpretations of the extent of global anoxic seafloor. Therefore, the carbon isotope record would appear to be decoupled from global redox changes, while local redox variability would still be plausible. However, in order for this mechanism to explain the global expression of the BACE, future work is needed to identify the drivers of local oxygenation and test whether upwelling of a deep dissolved organic carbon pool was synchronous across multiple paleocontinents.

Second, heightened volcanic activity could be the cause of global carbon isotope perturbation. Rift‐associated magmatic outgassing, combined with organic carbon or methane combustion, releases CO_2_ and can induce a negative δ^13^C excursion such as the BACE, which may also drive environmental changes and contribute to extinction events (e.g., Hodgin et al. 2021; Liyuan et al. 2021; Smith et al. 2023). Geochemical evidence for volcanism and/or hydrothermal fluxes could come from positive Eu anomalies (e.g., Bau 1991; Sverjensky 1984). The positive Eu anomalies (Eu/Eu* > 1.5) that occur at the nadir of the BACE at the Mount Dunfee and Cerro Rajón sections could reflect a regional input of hydrothermally sourced REE. This hydrothermal input could have resulted in the addition of low‐δ^13^C fluids to seawater along the southwestern Laurentian margin (see the crossplots of Eu anomaly and carbon isotope values in Figures S1 and S12). Rift‐related volcanic activity is recorded by metabasalt sills in the La Ciénega Formation and mafic–ultramafic flows in the overlying Cerro Rajón Formation (Barrón‐Díaz et al. 2019; Hodgin et al. 2021), while regionally, syndepositional basalts are also present in Stirling–Wood Canyon strata (Smith et al. 2023). Subsidence basin analysis, crustal uplift and volcaniclastic components with elevated Nd isotope ratios also support late Ediacaran to early Cambrian rifting in the western North American Cordillera, and potentially a hydrothermal input to local seawater at this time (Bond et al. 1985; Farmer et al. 2001; Levy and Christie‐Blick 1991). Overall, constraining the scale and duration of hydrothermal or volcanic activity is necessary to determine whether such a perturbation could have been reflected in a global carbon isotope shift.

The relationship between shallow marine oxygenation and early animal evolution remains debated. Our multi‐proxy study offers insights into how global and local redox variability may have influenced habitable shelf environments of early metazoans. The BACE—which occurred below the first appearance of complex bilaterian trace fossils (*Treptichnus pedum*)—was recorded under persistently reducing global conditions, without any global shifts in redox conditions. This observation could imply that the co‐occurrence of bilaterian behavioral evolution was not associated with a major increase in global ocean oxygenation. Instead, sufficient localized oxygen availability in shallow marine settings could have been crucial for early animal radiation (e.g., Alexander et al. 2025; Wood and Erwin 2018). Although early animals may have been tolerant of low‐oxygen conditions (Mills et al. 2014), dynamic redox conditions could have led to biological innovation by shaping behavioral complexity, physiological stress due to anoxia, and competition pressure from co‐habiting species (e.g., Childress and Seibel 1998; Twitchett 2007; Zhuravlev and Wood 2020), including the emergence of more complex burrowing behaviors. Thus, this study highlights the need to constrain local redox variability in shallow marine environments to understand how anoxic conditions exerted evolutionary pressure on the onset of complex metazoans.

## 6 Conclusions

Carbonate‐associated uranium isotopes from southwestern Laurentian strata indicate that the BACE, a global chemostratigraphic marker for the Ediacaran–Cambrian boundary, was not accompanied by widespread redox changes. Our new data, alongside a compilation of published uranium isotopes, indicate that global redox conditions remained anoxic with no observable changes to bottom‐water anoxia across the Ediacaran–Cambrian boundary and were not associated with the negative carbon isotope perturbation, highlighting the need to explore drivers of the BACE other than global marine oxygenation. The shallow marine environments represented by the carbonate strata of southwestern Laurentia experienced locally reducing conditions that varied within oxic–iodinous–manganous redox conditions. The dynamic redox variability of shallow environments is interpreted to reflect a regionally redox‐stratified ocean that is a consequence of low atmospheric oxygen levels during this time. Despite a more anoxic ocean compared to today, short‐lived and spatially limited oxygenation of the shallow oceans may have been critical in shaping ecological and evolutionary changes, including the increase in complexity of burrowing behaviors observed in the ichnofossil records directly above the BACE. In sum, our study highlights the necessity of multi‐proxy and multi‐site approaches for constraining heterogeneous marine redox conditions, which can reveal new insights into the correlation between carbon cycle perturbations and the marine redox state, as well as the relationship between environmental controls and early animal evolution.

## Funding

This work was supported by the National Science Foundation grants 2021324, 2021064, and 1923218, as well as by the Geological Society of America. Geological Society of America.

## Conflicts of Interest

The authors declare no conflicts of interest.

## Supporting information


**Figure S1:** Lithostratigraphy, δ^13^C chemostratigraphy, Eu anomaly (Eu/Eu*), and crossplots of δ^13^C and Ce anomaly against Eu anomaly from the Deep Spring Formation at Mount Dunfee (upper panel) and the La Ciénega Formation at Cerro Rajón (lower panel). The green line “1n” corresponds to the BACE nadir. Dark orange colors indicate Y/Ho > 36; light orange colors indicate Y/Ho between 25 and 36. If a correlation is statistically significant (*α* = 0.05), Spearman's rank correlation statistics are shown (purple = Deep Spring Formation and cyan = Cerro Rajón) as Spearman's *ρ*/*p*‐values.
**Figure S2:** Petrographic images in the left panel: the Deep Spring Formation, Mount Dunfee at 166, 332.5, 380.9, and 437.5 m and in the right panel: the La Ciénega Formation, Cerro Rajón at 7.5, 70, 113.7, 116, and 139 m. All images were taken with 2.5X and 5X objective lens under transmitted plane light unless another light source is specified. The scale bar on the bottom left corner of each image shows 500 μm, except for 380.9‐D and 113.7‐A with 1000 μm.
**Figure S3:** X‐Ray diffraction (XRD) analysis of a Cerro Rajón bulk powder sample at 113.7 m reveals semi‐quantitative mineral compositions of 78% dolomite, 9.3% quartz, 7.6% clinochlore, and 4.2% goethite.
**Figure S4:** Crossplots of Ce anomaly versus Fe and Mn concentrations and Bell Shape Index (BSI) (upper panel) and Y/Ho ratios versus Ce anomaly, Th and Zr concentrations (lower panel) from the Deep Spring Formation at Mount Dunfee (purple squares) and the La Ciénega Formation at Cerro Rajón (cyan triangles). All Spearman's *ρ* and *p*‐value are listed in Table S5.
**Figure S5:** Crossplot of uranium isotope values versus Mn concentration from the Deep Spring Formation at Mount Dunfee (purple squares) and the La Ciénega Formation at Cerro Rajón (cyan triangles). All Spearman's *ρ* and *p*‐value are listed in Table S5.
**Figure S6:** Crossplots of total rare earth element concentrations (ΣREE) versus Ce anomaly, Fe and Mn concentrations (upper panel) and elements representative of clays (i.e., Al, Th, and Zr) (lower panel) from the Deep Spring Formation at Mount Dunfee (purple squares) and the La Ciénega Formation at Cerro Rajón (cyan triangles). All Spearman's *ρ* and *p*‐value are listed in Table S5.
**Figure S7:** Comparison of acid, filter, and leaching REY protocols. Rare earth elements and yttrium (REY) distribution, normalized to post‐Archean Australian Shale (PAAS) are shown on a log scale. Protocol description: 0.3 N acetic acid = protocol A, 0.05 N hydrochloric acid = protocol B, 2% nitric acid = protocol C (see corresponding sample label in Table S3).
**Figure S8:** Crossplots of carbonate redox proxies (i.e., δ^238^U_carb_, Ce/Ce*, and I/(Ca + Mg)) versus Mg/Ca ratios from the Deep Spring Formation at Mount Dunfee (squares) and the La Ciénega Formation at Cerro Rajón (triangle; excluding two anomalously low δ^238^U_carb_ below −1‰ in the upper panel). Tan squares indicate the Dunfee Member and grey triangles indicate samples proximal (< 4 m) to interstratified metabasalt sills.
**Figure S9:** Crossplots of uranium isotope (δ^238^U_carb_) versus U/(Ca + Mg) ratio from the Deep Spring Formation at Mount Dunfee (purple squares = Esmeralda Member; tan squares = Dunfee Member), the La Ciénega Formation at Cerro Rajón (cyan triangles; excluding two anomalously low δ^238^U_carb_ below −1‰), and the Moosburg core from Southern Germany (circles; modified after Herrmann et al. (2018)). Spearman's rank correlation statistics are shown as Spearman's *ρ* and *p*‐values and colored by study sections.
**Figure S10:** Crossplots of δ^44^Ca data (modified from Lonsdale et al. n.d.) versus global and local seawater conditions of (A) δ^238^U_carb_ records, (B) I/(Ca + Mg) ratios, and (C) Ce anomalies from the Deep Spring Formation at Mount Dunfee (left panel) and the La Ciénega Formation at Cerro Rajón (right panel). Grayscale gradient corresponds to stratigraphic height. Colored rounded square and dashed arrows overlie predictions of initial seawater conditions (i.e., reduced vs. oxidized water) and early diagenetic transformations via various fluids (i.e., reduced, oxidized, and meteoric fluids) after a diagenetic modeling study (Lau and Hardisty 2022).
**Figure S11:** Crossplots of carbonate‐based redox proxies (i.e., δ^238^U_carb_, Ce/Ce*, and I/(Ca + Mg)). Purple squares indicate the Deep Spring Formation and cyan triangles indicate the La Ciénega Formation at Cerro Rajón. Colored regions: interpretations of global/local redox conditions, such as oxic, iodinous, manganous, and ferruginous–euxinic (also see Figure 2 and Figure 7). Dashed lines: red = modern global mean seawater δ^238^U (−0.379‰ ± 0.023‰; Kipp et al. 2022; Tissot and Dauphas 2015); green = modern oxygenated seawater for I/(Ca + Mg) ratio (2.6 μmol/mol; Glock et al. 2014; Lu et al. 2016); purple = positive Ce anomaly (Ce/Ce* > 1.2; Tostevin 2021).
**Figure S12:** Crossplots of Eu anomaly (Eu/Eu*) versus Ce anomaly (Ce/Ce*), carbon isotope (δ^13^C), total rare earth element (upper panel) and clay contents, such as Al, K, Zr, and Th (lower panel) from the Deep Spring Formation at Mount Dunfee (purple squares) and the La Ciénega Formation at Cerro Rajón (cyan triangles). Spearman's rank correlation statistics are shown (purple = Deep Spring Formation and cyan = Cerro Rajón) as Spearman's *ρ* and *p*‐values.

**Table S1:** Geochemical results from the Deep Spring Formation, Mount Dunfee, USA; BDL, Below Detection Limit; NA, not available/no measurement.
**Table S2:** Geochemical results from the La Ciénega Formation, Cerro Rajón, Mexico; BDL, Below Detection Limit; NA, not available/no measurement.
**Table S3:** Acid comparison results (varying acid types, digestion protocol, and filter usage); BDL, Below Detection Limit; rep, sample replica.
**Table S4:** Compilation of dominant redox conditions from the marine carbonate and shale studies during late Ediacaran and early Cambrian (shown in Figure 7).
**Table S5:** Spearman's rank correlation coefficients (*ρ*) and *p*‐values calculated to test for the influence of diagenetic indicators. Statistically significant relationships are bolded and italicized (*α* = 0.05).

## Data Availability

The data that supports the findings of this study are available in the Supporting Information—S1 of this article.
