# Supplementary material for: Localized and Transient Oxygenation of Shallow Oceans of Southwestern Laurentia at the Ediacaran–Cambrian Boundary
Source: Geobiology. 2025 Dec 30;24(1):e70039. doi: 10.1111/gbi.70039 (PMC12754582; doi:10.1111/gbi.70039)
Supplement: Supplementary file 1 — Figure S1: Lithostratigraphy, δ13C chemostratigraphy, Eu anomaly (Eu/Eu*), and crossplots of δ13C and Ce anomaly against Eu anomaly from the Deep Spring Formation at Mount Dunfee (upper panel) and the La Ciénega Formation at Cerro Rajón (lower panel). The green line “1n” corresponds to the BACE nadir. Dark orange colors indicate Y/Ho > 36; light orange colors indicate Y/Ho between 25 and 36. If a correlation is statistically significant (α = 0.05), Spearman's rank correlation statistics are shown (purple = Deep Spring Formation and cyan = Cerro Rajón) as Spearman's ρ/p‐values. Figure S2: Petrographic images in the left panel: the Deep Spring Formation, Mount Dunfee at 166, 332.5, 380.9, and 437.5 m and in the right panel: the La Ciénega Formation, Cerro Rajón at 7.5, 70, 113.7, 116, and 139 m. All images were taken with 2.5X and 5X objective lens under transmitted plane light unless another light source is specified. The scale bar on the bottom left corner of each image shows 500 μm, except for 380.9‐D and 113.7‐A with 1000 μm. Figure S3: X‐Ray diffraction (XRD) analysis of a Cerro Rajón bulk powder sample at 113.7 m reveals semi‐quantitative mineral compositions of 78% dolomite, 9.3% quartz, 7.6% clinochlore, and 4.2% goethite. Figure S4: Crossplots of Ce anomaly versus Fe and Mn concentrations and Bell Shape Index (BSI) (upper panel) and Y/Ho ratios versus Ce anomaly, Th and Zr concentrations (lower panel) from the Deep Spring Formation at Mount Dunfee (purple squares) and the La Ciénega Formation at Cerro Rajón (cyan triangles). All Spearman's ρ and p‐value are listed in Table S5. Figure S5: Crossplot of uranium isotope values versus Mn concentration from the Deep Spring Formation at Mount Dunfee (purple squares) and the La Ciénega Formation at Cerro Rajón (cyan triangles). All Spearman's ρ and p‐value are listed in Table S5. Figure S6: Crossplots of total rare earth element concentrations (ΣREE) versus Ce anomaly, Fe and Mn concentrations (upper panel) and eleme [file GBI-24-e70039-s001.docx]

# Supplementary material (Data S1)

## Petrographic analysis

This study utilizes outcrop samples for geochemical analyses. Weathering as well as syn- and other post-depositional processes could have impacted on the original carbonate geochemistry. Prior to sample powdering, we removed surficial rinds with a Dremel tool. During sample powdering, visible veins, fractures, and regions indicative of alteration, such as recrystallization, oxidized coloration, and secondary minerals, were avoided. Nine representative thin sections were prepared by Wagner Petrographic. Four thin sections were made from samples of the Deep Spring Formation at Mount Dunfee at stratigraphic heights of 166, 332.5, 380.9, and 437.5 m, and five thin sections were made from samples of the La Ciénega Formation at Cerro Rajón at stratigraphic heights of 7.5, 70, 113.7, 116, and 139 m. Carbonate fabric, mineralogy, and diagenetic features were observed under plane and cross-polarized light and imaged using an Axio Imager2 microscope and under reflective light using a Keyence microscope at Pennsylvania State University.

At Mount Dunfee (Figure S2, left panel), the carbonate fabrics show mixed recrystallization style, ranging from micrite, microspar, and spar calcites. We did not observe an apparent change in crystal size or other textures with stratigraphic height, although the sample at 437.5 m is overall more coarsely crystallized than the sample at 166 m (Figure S2 437.5 m-A; 116 m-A, respectively). We noted that this succession contains the highest diversity of diagenetic features, including calcite veins (Figure S2 116 m-B) and stylolites (Figure S2 332.5 m-A, B). A few samples contain non-carbonate phases, such as detrital quartz (Figure S2 380.9-A, B) and opaque square crystals (likely iron oxide minerals) (Figure S2 380.9 m-C). Under reflective light, additional textures such as microbial-like structure, trapped detrital quartz, and dolomitic rhombs are revealed (Figure S2 380.9 m-D). Relict organic matter appears as brown, opaque, non-structured masses under transmitted light (Figure S2 437.5 m-B). The diverse mineralogy may reflect a shallow marine environment with periods of increased siliciclastic input.

At Cerro Rajón (Figure S2, right panel), carbonate fabrics contain dolomitic microspar, with several mm-scale regions of micritic fabric (Figure S2 7.5 m-A). The preservation of micrite and peloids suggests fabric-retentive dolomitization. Dolomite crystal size appears to increase with stratigraphic height between 7.5 m and 70 m, with interlocking crystals observed at 139 m (Figure S2 7.5 m-B; 70 m-A; 139 m-A). Relict organic matter is preserved at lower stratigraphic heights (Figure S2 7.5 m-C, D). Due to the low sampling resolution, we cannot confidently identify a shift from fabric retentive to fabric destructive dolomitization across this stratigraphic interval. We also note that the sample at 113.7 m near the BACE nadir is distinct in color and mineralogy (Figure S2 113.7 m-A). A nearby sample at 116 m contains opaque black to dark red minerals that may reflect the presence of iron oxides (Figure S2 116 m-A). X-ray diffraction analysis (XRD) of the sample at 113.7 m indicates that the mineralogy is dominantly dolomite, with minor quartz, goethite, and clinochlore (Figure S3). The mineral composition of this sample may suggest a paragenetic sequence with relatively early dolomitization, followed by the precipitation of hydrothermal minerals such as clinochlore (Dekov et al., 2008).

Petrographic analysis indicates varying degrees of recrystallization, without a clear relationship between crystal size and stratigraphic changes in either studied succession. The preservation of micritic fabric in the La Ciénega Formation suggests marine carbonate geochemistry and early marine diagenesis. The Deep Spring samples contain both micrite and recrystallized textures, as well as a diverse mineralogy. Importantly, our petrographic analyses were not intended to document the entirety of diagenetic alteration within these sections, but rather to better characterize the paragenetic features present in our samples to inform geochemical interpretations.

## The Rare Earth elements and Yttrium (REY) patterns

The REY patterns and trace elemental concentrations of carbonates can provide insight into whether carbonate geochemistry broadly records seawater chemistry. Carbonates precipitated from modern seawater are characterized by low total concentrations of rare earth elements (ΣREE) and low bell-shaped index (BSI) values. High BSI can indicate contamination from phosphate and/or iron oxide sources, which result in elevated concentrations of the middle rare earth elements (MREE) (Bau & Dulski, 1996; Tostevin et al., 2016a):

$$BSI= \frac{2*({[Sm]}_{SN}+{[Gd]}_{SN}+{[Dy]}_{SN})/3}{({[La]}_{SN}+{[Pr]}_{SN}+{[Nd]}_{SN})/3+({[Ho]}_{SN}+{[Er]}_{SN}+{[Tm]}_{SN}+{[Yb]}_{SN}+{[Lu]}_{SN})/5}$$

All BSI values are consistently low at both sites (Figure S4 upper panel), indicating limited contribution from Fe and Mn (oxyhydr)oxide or phosphate minerals. Contamination from oxide minerals should result in positive Ce/Ce* and reduced Y/Ho ratios. Because the samples with high concentrations of Fe and Mn also have low BSI, we do not interpret significant oxide contributions of REE within our data. We also do not observe correlations between Fe and Mn concentrations with the Ce anomaly at either study site (Figure S4 upper panel).

The modern seawater REY pattern is characterized by depleted light rare earth elements (LREE: La, Ce, Pr, Nd) and enriched heavy rare earth elements (HREE: Ho, Er, Tm, Yb, Lu). Conversely, MREE or LREE enrichments suggest diagenetic alteration due to early diagenetic mobilization of REE that deviates from the pattern of modern oxygenated seawater (Zhang & Shields, 2023). Both carbonate successions do not typically exhibit MREE nor LREE enrichments (Figure 4). A few exceptions include six Deep Spring samples with HREE depletion (i.e., Pr_SN_/Yb_SN_ > 0.8) and HREE depletion in Units 2 and 4 in the La Ciénega Formation. The Dy_SN_/Sm_SN_ ratios, which are used to indicate MREE enrichments, are ~1.4 (average, n=22) and ~1.2 (average, n=28) in the Deep Spring and La Ciénega formations, respectively (Table S1 and Table S2). These ratios may suggest a deviation from a typical seawater REY distribution. Overall, the REY patterns from the Deep Spring and La Ciénega formations suggest the potential of alteration due to REY remobilization. However, this mobilization should not impact Ce/Ce* interpretations unless incorporated with substantial REE carrier fluxes, such as Fe-Mn oxides, which we did not observe any trend with low BSI values.

The seawater REY distribution contains a small positive Eu anomaly (≤ 1.5) and a high Y/Ho ratio (>36), but these characteristics are not consistently present in the La Ciénega Formation. A high Eu anomaly (Eu/Eu*) can indicate contributions from hydrothermal fluids, because reducing conditions permit Eu mobilization and induce Eu enrichments relative to its neighboring REE (Meyer et al., 2012; Mitra et al., 1994; Olivarez & Owen, 1991; Tostevin et al., 2016a). Modern carbonates with no hydrothermal influence contain Eu/Eu* values that range from ~1 to ~1.5 (Tostevin et al., 2016c). Both Mount Dunfee and Cerro Rajón sections contain multiple samples with Eu/Eu* anomalies slightly above 1.5, reflecting minor Eu enrichments. The average Eu/Eu* in the La Ciénega Formation is 1.53 (n=28), and the maximum value is less than 2 (Figure S1 and Table S2). In the Deep Spring Formation, the maximum Eu/Eu* reaches 2.14 and the average is 1.50 (n=22) (Figure S1 and Table S1). These values are not significantly different from other coeval carbonate records with Eu/Eu* ranging from 0.9 to 2.4 (e.g., Kamber and Webb, 2001; Ling et al., 2013; Chen et al., 2014; Hohl et al., 2017). Therefore, the Eu anomaly in both the Mount Dunfee and Cerro Rajón sections are not anomalous for carbonates from this time interval, and we do not interpret these values as indicative of a non-seawater influence.

The co-occurrence of negative Ce/Ce*, positive Eu/Eu*, and the BACE in the La Ciénega Formation may reflect unusual hydrothermal activity that locally enhanced Eu input into seawater during that period. First, negative Ce and positive Eu anomalies are expected to be evolved separately, because a negative Ce anomaly indicates oxidizing, low-temperature conditions (Elderfield & Greaves, 1982; Loges et al., 2012; Seto & Akagi, 2008), and a positive Eu anomaly indicates reducing, high-temperature fluids (Bau, 1991; Sverjensky, 1984). Modern anoxic seawater can contain both positive Eu/Eu* (e.g., Bannock Basin in eastern Mediterranean Sea; Schijf et al., 1995) and no Eu anomaly (e.g., the Cariaco Trench in the Venezuelan shelf; de Baar et al., 1988). In other words, Eu anomaly is not a reliable indicator for basinal anoxia. Therefore, we did not interpret the positive Eu anomalies to reflect seawater redox conditions.

Yet, the crossplots of Eu anomaly and carbon isotope in Figure S1 display a statistically significant inverse correlation at both study sites (Spearman’s rank, *p*-values: 0.004 in the Deep Spring Formation and 0.002 in the La Ciénega Formation). If the BACE represents a global carbon cycle perturbation, Eu anomaly with a shorter residence time in seawater would likely record local/ diagenetic processes rather than a global signal. However, we speculate that hydrothermal input could act as a common driver within our studied strata, because we observe evidence of local magmatism, specifically metabasalt sills in the La Ciénega Formation and mafic–ultramafic flows in the overlying Cerro Rajón Formation Formation (Barrón-Díaz et al., 2019; Hodgin et al., 2021) and the deposition of rifting and post-rifting transition in the western North American Cordillera during the latest Proterozoic–early Paleozoic boundary (Bond et al., 1985; Farmer et al., 2001; Levy & Christie-Blick, 1991).

In addition to hydrothermal or volcanic processes, we also explore other potential mechanisms that could result in a positive Eu anomaly (Eu/Eu* > 1.5) including: high-temperature alteration, analytical artefacts, facies/lithologic factors, Eu remobilization, and excessive Eu input from weathering:

1. High-temperature alteration – Europium is reduced to Eu^2+^ as temperature increases to 250−600 °C, such as in a mid-ocean ridge system (Bau, 1991; Sverjensky, 1984), and both Eu and Ce reduction can also occur via hydrothermal reactions (Lee et al., 2025). Although the La Ciénega Formation is closely associated with metabasalt sills, which could have resulted in high-temperature alteration, this mechanism is inconsistent with the co-occurrence of negative Ce anomalies, indicating the presence of oxidized Ce^4+^. In addition, the majority of our La_SN_/Lu_SN_ ratios are below 1, indicating neutral to mildly basic fluids (Bau, 1991). Basic conditions favor REE complexation that stabilize Eu^3+^ and would not produce positive Eu anomalies. Therefore, the observed Eu anomalies are unlikely produced through this process.
2. Analytical artefact – Eu enrichment can be an analytical artifact if BaO+ interferences were not corrected for during ICP-MS analyses (Shields & Stille, 2001). After correction for BaO^+^ interference, only a single sample of Eu/Eu* above 1.5 in the La Ciénega Formation contained Ba/Eu ratios greater than 1000. Therefore, the observed positive Eu anomalies likely result from natural variability.
3. Facies/lithologic control – It is possible that dolomitization and associated alteration resulted in enrichment of Eu (Hu et al., 2019). However, the presence of positive Eu anomalies in both limestone and dolostone samples suggests that the Eu anomaly is unlikely controlled by lithology.
4. Eu remobilization – Selective remobilization by diagenetic fluids can be characterized by MREE enrichment, which potentially could result in a positive Eu anomaly after exchanging with hydrothermal fluids (Bau et al., 2014; Zhang & Shields, 2023). However, we do not observe MREE enrichment with positive Eu/Eu* in our studied strata.
5. Excessive Eu input from weathering – Previous studies have shown that Eu^2+^ is preferentially released from feldspar during weathering (Kamber et al., 2005; Nozaki et al., 2000). Sedimentary rocks that are composed of feldspathic and clay components—such as those locally present in nearby sandstones of the Wood Canyon Formation (Levy & Christie-Blick, 1991)—could increase Eu levels in carbonate rocks if the weathering processes release trace elements and are transported into and come into contact with carbonate depositional settings. We performed a sequential digestion to target carbonate phases while avoiding clay contaminations (Tostevin et al., 2016b), and we found no consistent correlation between positive Eu anomalies and total REE concentrations in both sections (Figure S12). Further, the correlation between clay content (e.g., Al and K) and the Eu anomaly is only observed in the La Ciénega Formation and is absent in the Deep Spring Formation (Figure S12). Therefore, we cannot conclude that excessive Eu input was a common driver for positive Eu anomalies in both successions during the BACE nadir.

Therefore, none of these mechanisms can account for the coincidence of positive Eu anomalies and the negative carbon excursion at both sites.

Clay contamination can result in high ΣREE because REY readily sorb to clay minerals (Bau et al., 1996; Nothdurft et al., 2004; Taylor & McLennan, 1985). The ΣREE values are generally higher in the Deep Spring Formation at Mount Dunfee with an average of 53.2 ppm (n=22) compared to 15.8 ppm (n=28) in the La Ciénega Formation at Cerro Rajón (Table S1 and Table S2). The crossplots of clay and detrital content (i.e., Al, Th, and Zr) from the Deep Spring Formation show a significant correlation with the ΣREE values (Figure S6). We hypothesize that the Deep Spring Formation may record distinct carbonate geochemistry due to riverine mixing. Specifically, a few Deep Spring samples with elevated Al and Th concentrations tend to display low Y/Ho ratios, no Ce anomalies, and positive Eu anomalies that are likely consistent with REY addition from non-seawater sources.

A pronounced Y anomaly (i.e., related to Y/Ho > 36) is another characteristic of seawater. The La Ciénega Formation has typically high Y/Ho ratios than 36, whereas the Deep Spring Formation has low ratios (average Y/Ho ~ 25). The absence of pronounced Y anomalies in the Deep Spring Formation may suggest input from non-carbonate sources, such as freshwater mixing and/or local depletion in Y relative to Ho. The potential of non-carbonate contamination is unlikely due to our acid leaching (discussed below). Further, sedimentary structures and fossils at this site are interpreted to represent an open marine environment (Smith et al., 2016). The crossplots of Y/Ho against Ce anomaly and elements representative of terrestrially derived clay do not suggest that inputs from siliciclastic material affected Y/Ho ratios and Ce anomalies at both sections (Figure S4 lower panel). However, due to generally high ΣREE values and low Y/Ho ratios in the Deep Spring Formation, we conservatively do not interpret local seawater redox conditions using the Ce anomaly proxy at the Mount Dunfee section. In contrast, carbonate samples from the La Ciénega Formation are interpreted as preserving seawater Ce anomalies based on Y/Ho ratios and low BSI, ΣREE, and Eu anomalies.

## REY acid digestion comparison test

We performed a sample preparation test to evaluate whether our REY leaching procedure can adequately avoid contamination from non-carbonate phases. We varied the use of cleaning reagent (1 M ammonium acetate), the acid used for digestion (0.3 N acetic acid, 0.05 N hydrochloric acid, 2% nitric acid), and whether a filtration step was included.

In protocol A, ammonium acetate cleaning and acetic acid digestion follows the protocol from Cao et al. (2020). In protocol B, hydrochloric acid digestion follows the protocol from Kalderon-Asael et al. (2021), which is designed for carbonate lithium isotopes that are particularly sensitive to clay contamination. In protocol C, the carbonate sequential dissolution with nitric acid follows the protocol from Tostevin et al. (2016b). All tests were performed on aliquots of homogenous powder from one sample each from both sections.

In all protocols, carbonate powder samples were cleaned using 2.5 mL 1 M ammonium acetate (pH of 6.9) and placed in a sonicator bath for 30 minutes. The samples were centrifuged, then the supernatant was removed via pipetting, and the remaining residue was rinsed with 1.25 mL MilliQ water and discarded. The ammonium acetate addition was repeated due to high carbonate content (> 85 wt.%) and rinsed twice with MilliQ water to avoid carry-over effects on REY. A few control samples were cleaned with ~ 1–2 mL MilliQ water and pipetted to discard the supernatant after vortex and centrifugation.

For acetic acid digestion in protocol A, 1.25 mL of 0.3 N acetic acid was added to 50 mg of carbonate powder and allowed to react for 30 minutes. For hydrochloric acid digestion in protocol B, 3.33 mL of 0.05 N hydrochloric acid was added to 50 mg of carbonate powder and the supernatant was collected after a sequential digestion for 4 hours, 2 hours, and 10 minutes. For nitric acid digestion of dolomite-rich samples in protocol C, 0.723 mL of 2% nitric acid was added to 50 mg of sample powder and placed on a shaker table for 20 minutes. For nitric acid digestion of calcite-rich samples in protocol A, 0.666 mL of 2% nitric acid was added to 50 mg sample powder, discarded after 20 minutes on a shaker table. The sample was then rinsed three times with MilliQ water, then 1.332 mL of 2% nitric acid was added and placed on a shaker table for 20 minutes.

For all protocols, a filtration step was included during collection of the supernatant for all acids tested. The samples were passed through a Thermo Scientific Titan3 Polyethersulfone syringe filter (pore size of 0.22 μm), which had been rinsed with the same acid used to leach each sample.

For protocols A and B, samples digested with hydrochloric and acetic acids were placed on a hotplate until completely dried. Then, 0.5–1 mL of concentrated nitric acid was added to transform the matrix to nitric acid. All samples in all protocols were diluted in 2% nitric acid and analyzed on a Thermo Fisher Scientific iCAP RQ ICP-MS in the Laboratory for Isotopes and Metals in the Environment (LIME) facility at Pennsylvania State University.

The acid digestion comparison results are reported in Figure S7 and Table S3. We found that the different acid leaches resulted in varying dissolution of clay elements. Specifically, the nitric acid leaches resulted in higher Th whereas hydrochloric acid leaches resulted in higher Zr levels (Table S3). In contrast, acetic acid leaches resulted lower clay contribution, indicated by lower concentrations of Al and ΣREE. The filtration step also reduced Al levels but did not significantly impact Zr and Th concentrations (Table S3). In general, the use of 2% nitric acid could result in higher Al, Zr, Th, and ∑REE compared to the use of hydrochloric acid and acetic acid. However, similar Ce and Eu anomalies, Y/Ho ratios, and bell-shaped index values were reproducible for all samples between the three protocols despite the use of different cleaning steps, acids, and filtration. This result suggests that local redox conditions from the Ce anomaly are not influenced by the acid and protocol employed. Additionally, the consistent Y/Ho ratios across the acid digestion comparison suggest this proxy can be a tracer for seawater signals. Consistent BSI values also indicate limited contribution of Fe and Mn (oxyhydr)oxide or phosphate minerals by the digestion step. Although interpretations of clay concentrations could vary depending on the acid type, our selected leaching protocol using 2% nitric acid without filtration (protocol C) is expected to reflect the carbonate Ce anomaly in calcite and dolomite samples.

## Calcium isotope constraints on marine diagenesis

Calcium isotopes (δ^44/40^Ca) aid in distinguishing early marine diagenetic regimes and evolving pore fluid alteration and quantify the extent of recrystallization (e.g., Ahm et al., 2018; Higgins et al., 2018; Lau & Hardisty, 2022). Measured δ^44/40^Ca values from these samples suggest dominantly seawater-buffered conditions in both the Deep Spring and La Ciénega formations, based on high δ^44/40^Ca (> –0.8‰) and low Sr/(Ca+Mg) (< 0.5 mmol/mol) (Lonsdale, 2025; Lonsdale et al., *accepted*).

We compared these δ^44/40^Ca data with our redox proxies to assess early diagenetic overprinting in our paleoredox records using diagenetic model predictions (Lau & Hardisty, 2022). The δ^238^U_carb_ shows a large range from ~0.1 to –0.8‰, whereas δ^44/40^Ca data span –1.6‰ to –0.7‰ and –1.2‰ to –0.8‰ in the Deep Spring and La Ciénega formations, respectively (Figure S10 A). The crossplot of δ^44/40^Ca versus δ^238^U_carb_ (Figure S10 A) shows a unique relationship that does indicate diagenetic alteration on trace element (and specifically uranium composition) for upper Dunfee Member samples. The four samples from the Dunfee Member show increasing δ^44/40^Ca (–1.6‰ to –1.2‰) with increasing δ^238^U_carb_ values that are much higher than continental inputs. This trajectory matches the model prediction for diagenetic recrystallization with reducing seawater (see red dashed line in Figure S10 A). Therefore, the change in δ^238^U_carb_ of these samples is unlikely recording a change in seawater but instead diagenetic alteration associated with increasing recrystallization. Therefore, we excluded the Dunfee Member samples from the δ^238^U_carb_ compilation in Figure 8. Beyond these samples, no clear Ca and U isotope trends follow the modeled diagenetic trajectories, in the Esmeralda Member and the La Ciénega Formation. The I/(Ca+Mg) ratios are consistently low and uncorrelated with δ^44/40^Ca data at both sections (Figure S10 B). Given the prevalence of iodide in reducing pore fluids, it is unclear whether samples with I/(Ca+Mg) < 1.2 μmol/mol and below detection reflect diagenetic overprinting and/or low oxygen levels in surface seawater (Hardisty et al., 2017; Hashim et al., 2022; Lau & Hardisty, 2022). In the Deep Spring Formation, the broader δ^44/40^Ca range may indicate variable diagenesis, suggesting below detection I/(Ca+Mg) ratios could result from diagenetic alteration with locally reducing seawater. The Ce anomalies and δ^44/40^Ca data also do not fall along modeled diagenetic trajectories (Figure S10 C), indicating that early pore fluid diagenesis did not drive observed redox changes. We note that a few La Ciénega samples with negative Ce anomalies align with initially oxidizing seawater conditions. This result further supports our interpretation that the local seawater was initially oxygenated prior to the early marine diagenesis (and also not controlled by meteoric mixing fluid) during the BACE nadir in the Cerro Rajón section. Overall, while marine diagenesis varied, redox proxy records are not fully overprinted by early seawater diagenetic alteration.

## Statistical analysis

We evaluated correlations between geochemical data using Spearman’s rank correlation. This test is appropriate because non-linear trends are expected in our data, such as for diagenesis (e.g., Mn/Sr; Brand and Veizer, 1980). The Spearman’s rank correlation was calculated by function *cor.test* in R. We used Spearman’s rank correlation coefficient, ρ, to assess the significance and direction (i.e., positive or negative) of a correlation. The magnitude of the coefficient determines if a correlation is weak (< 0.1 to 0.3), moderate (0.4 to 0.6), or strong (0.7 to > 0.9). We chose the significance level (α) of 0.05 to represent the threshold where a correlation is statistically significant. In Figures 5, 6, S1, S4, S6, S9, and S12, ρ and *p*-values are shown for statistically significant correlations. Statistically significant correlations are bolded and italicized in Table S5. In addition, we used a t-test to compare two data sets with normal distribution to determine a significant difference between them. We compared the means of uranium isotope values from the Dunfee Member and Esmeralda Member of the Deep Spring Formation. The t-test provides a small *p*-value of 0.0174, indicating a statistical significance between these two members.

# Supplementary figures

**~~
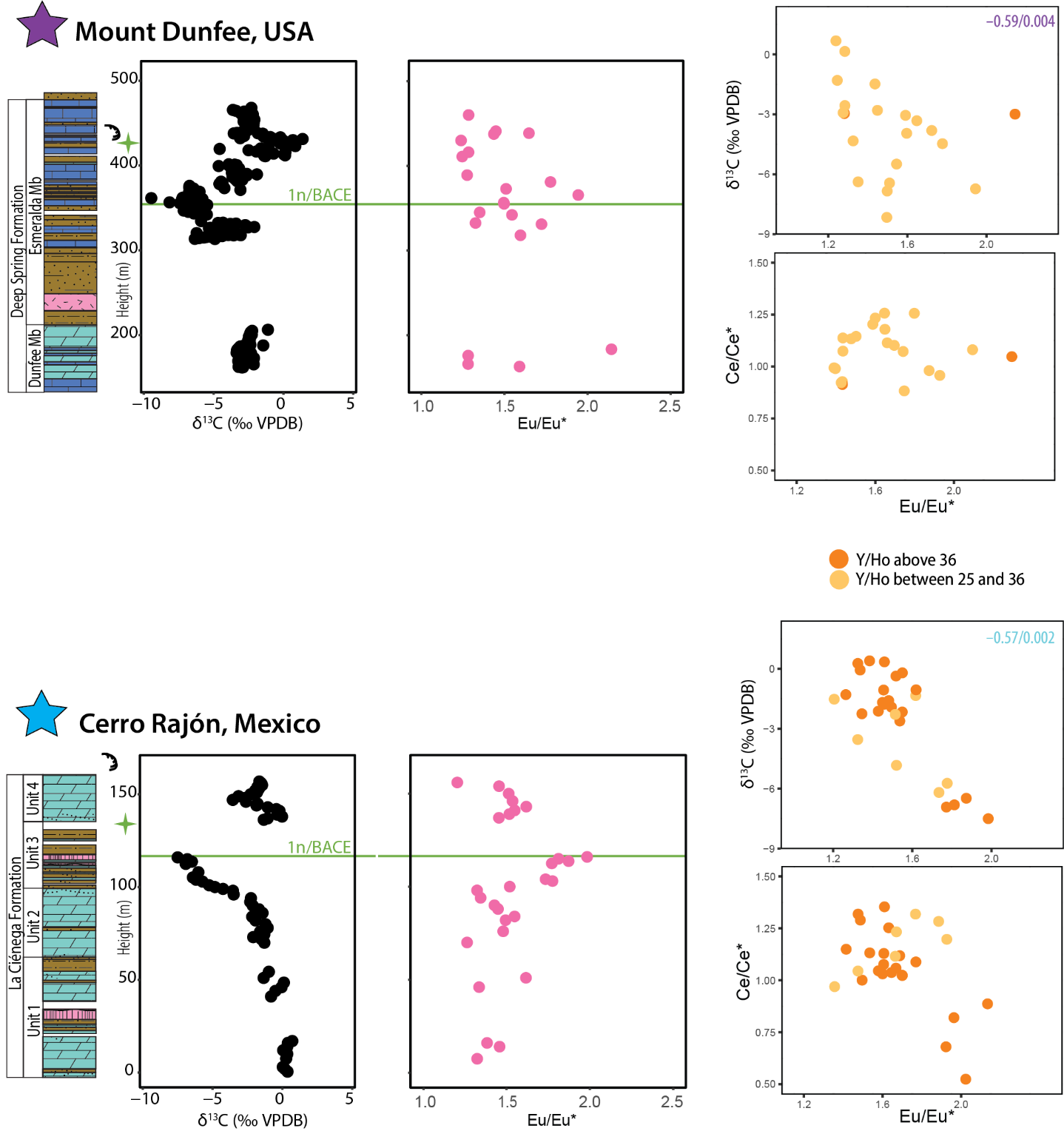
~~**

**Figure S1** – Lithostratigraphy, δ^13^C chemostratigraphy, Eu anomaly (Eu/Eu*), and crossplots of δ^13^C and Ce anomaly against Eu anomaly from the Deep Spring Formation at Mount Dunfee (upper panel) and the La Ciénega Formation at Cerro Rajón (lower panel). The green line “1n” corresponds to the BACE nadir. Dark orange colors indicate Y/Ho > 36; light orange colors indicate Y/Ho between 25 and 36. If a correlation is statistically significant (α=0.05), Spearman’s rank correlation statistics are shown (purple = Deep Spring Formation and cyan = Cerro Rajón) as Spearman’s ρ / *p*-values.

**~~
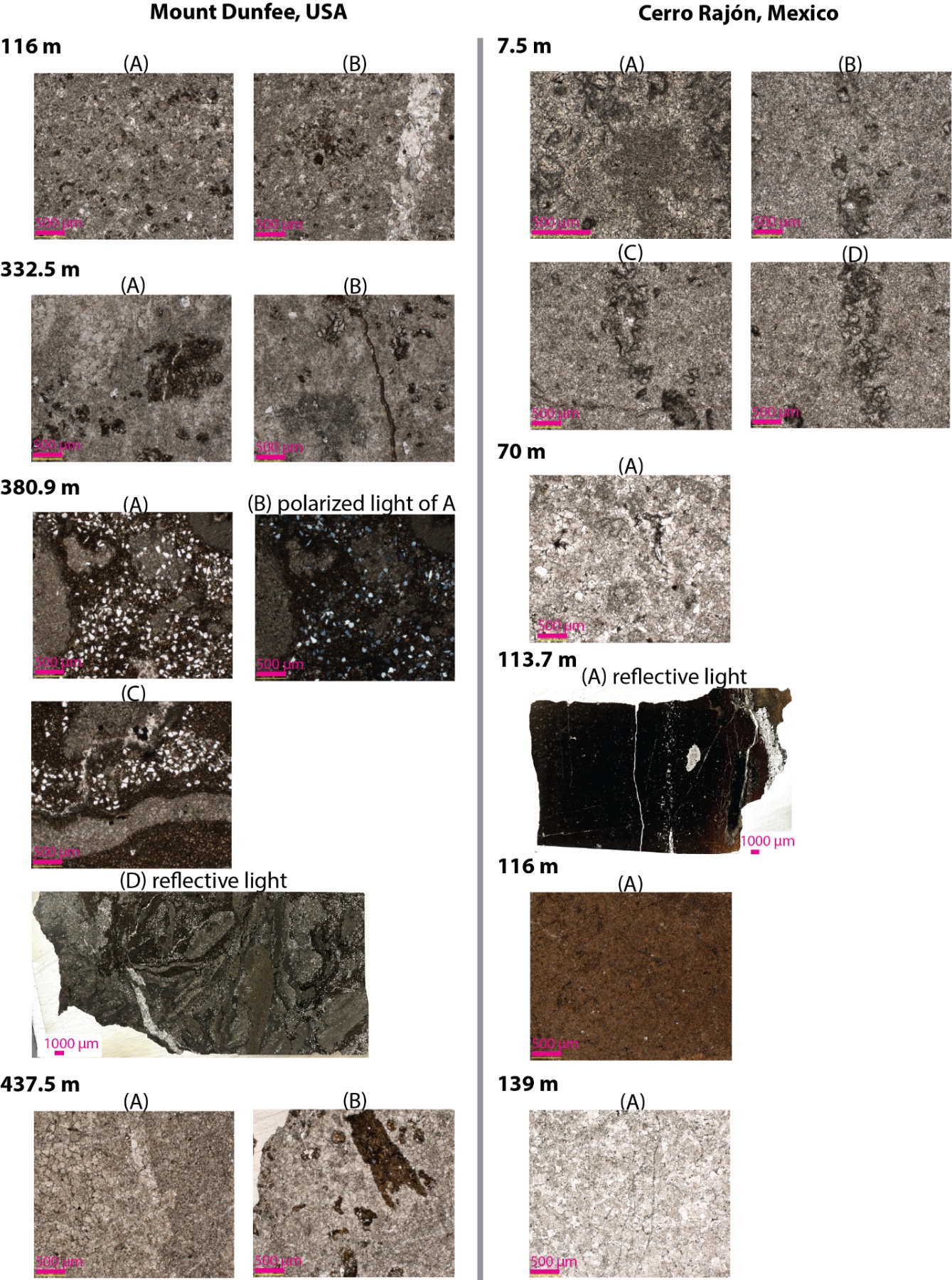
~~**

**Figure S2** – Petrographic images in the left panel: the Deep Spring Formation, Mount Dunfee at 166, 332.5, 380.9, and 437.5 m and in the right panel: the La Ciénega Formation, Cerro Rajón at 7.5, 70, 113.7, 116, and 139 m. All images were taken with 2.5X and 5X objective lens under transmitted plane light unless another light source is specified. The scale bar on the bottom left corner of each image shows 500 µm, except for 380.9 m-D and 113.7 m-A with 1000 µm.


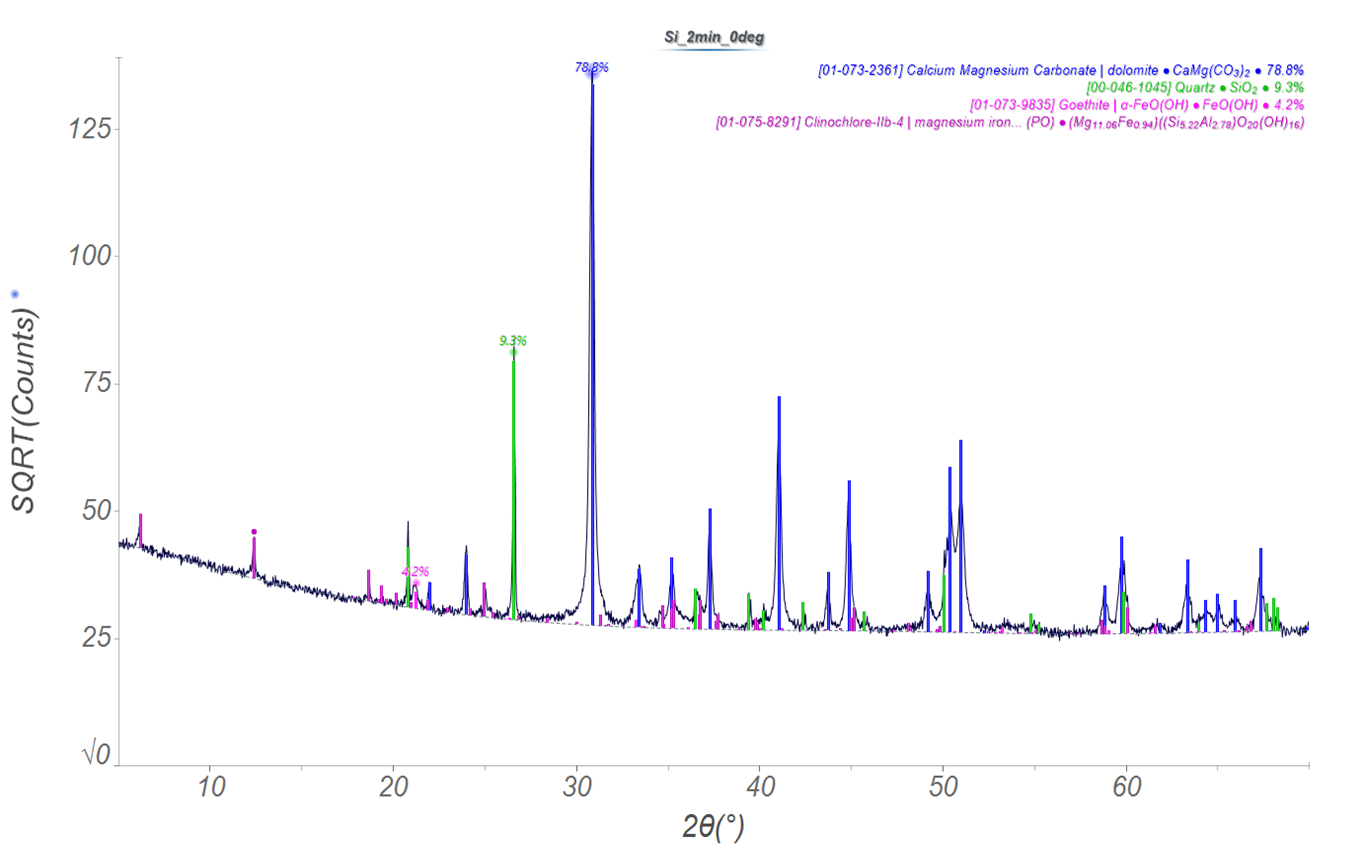


**Figure S3** – X-Ray diffraction (XRD) analysis of a Cerro Rajón bulk powder sample at 113.7 m reveals semi-quantitative mineral compositions of 78% dolomite, 9.3% quartz, 7.6% clinochlore, and 4.2% goethite.


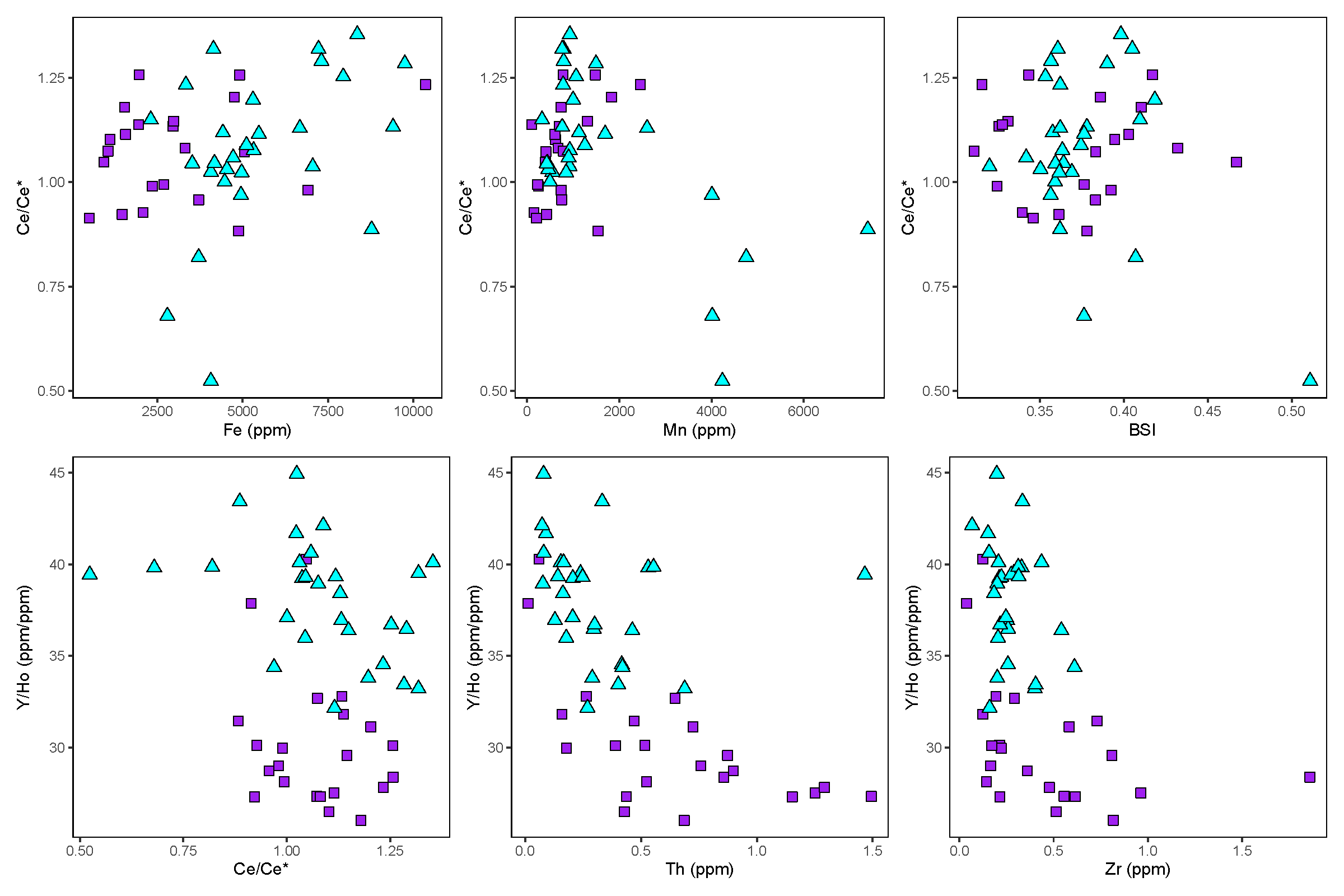


**Figure S4** – Crossplots of Ce anomaly versus Fe and Mn concentrations and Bell-Shape Index (BSI) (upper panel) and Y/Ho ratios versus Ce anomaly, Th and Zr concentrations (lower panel) from the Deep Spring Formation at Mount Dunfee (purple squares) and the La Ciénega Formation at Cerro Rajón (cyan triangles). All Spearman’s ρ and *p*-value are listed in Table S5.


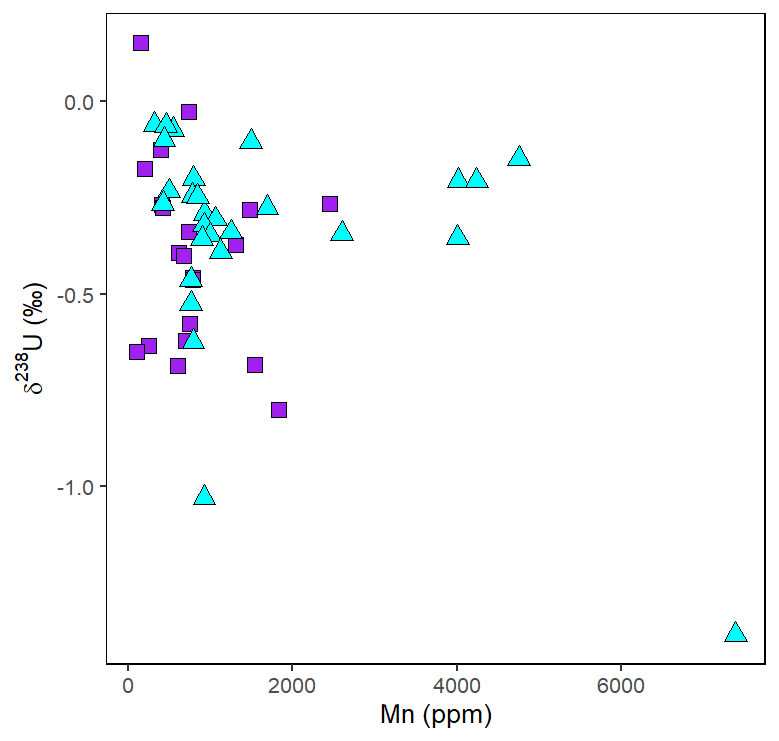


**Figure S5** – Crossplot of uranium isotope values versus Mn concentration from the Deep Spring Formation at Mount Dunfee (purple squares) and the La Ciénega Formation at Cerro Rajón (cyan triangles). All Spearman’s ρ and *p*-value are listed in Table S5.


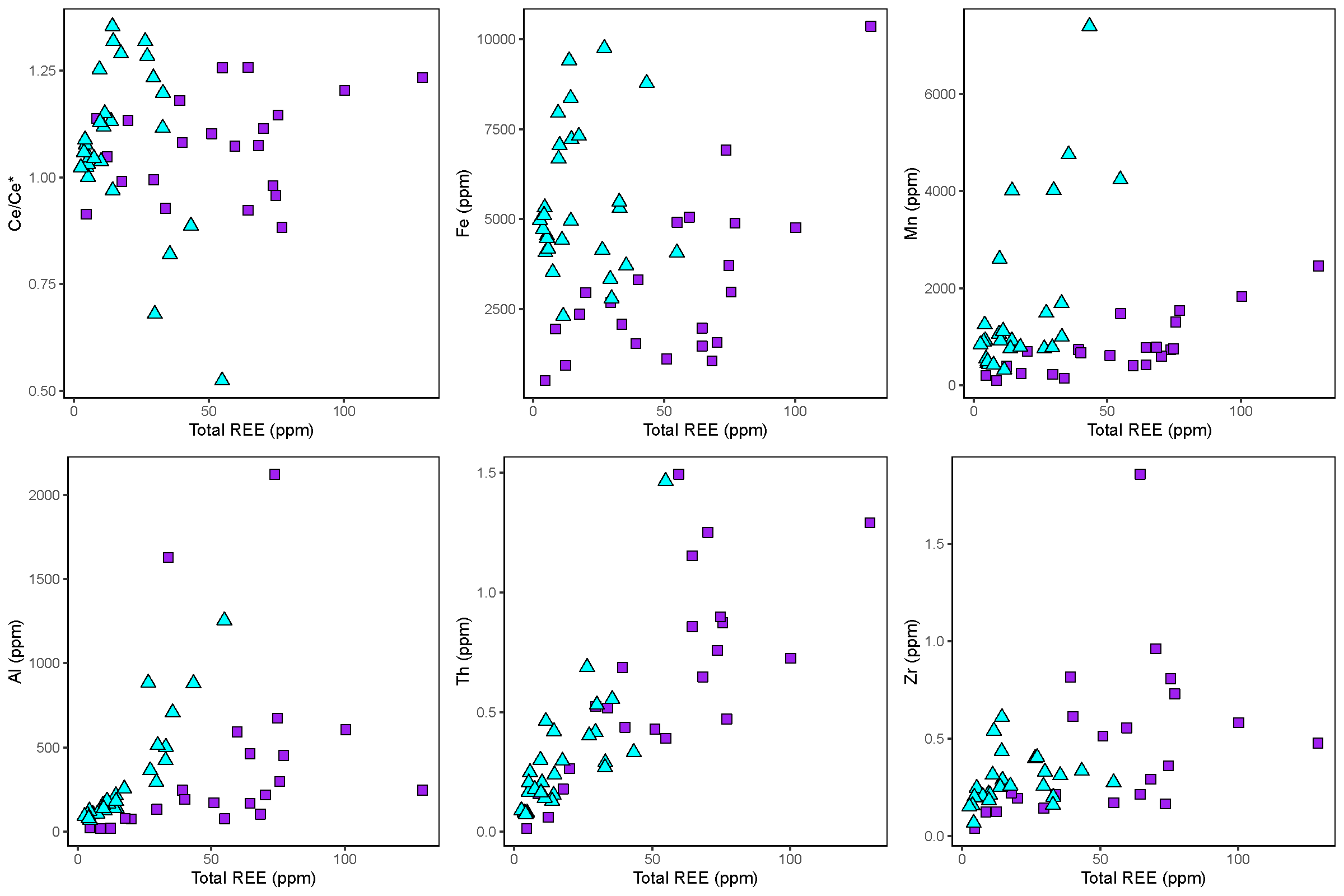


**Figure S6** – Crossplots of total rare earth element concentrations (ΣREE) versus Ce anomaly, Fe and Mn concentrations (upper panel) and elements representative of clays (i.e., Al, Th, and Zr) (lower panel) from the Deep Spring Formation at Mount Dunfee (purple squares) and the La Ciénega Formation at Cerro Rajón (cyan triangles). All Spearman’s ρ and *p*-value are listed in Table S5.

**Figure S7** – Comparison of acid, filter, and leaching REY protocols. Rare earth elements and yttrium (REY) distribution, normalized to post-Archean Australian Shale (PAAS) are shown on a log scale. Protocol description: 0.3 N acetic acid = protocol A, 0.05 N hydrochloric acid = protocol B, 2% nitric acid = protocol C (see corresponding sample label in Table S3).


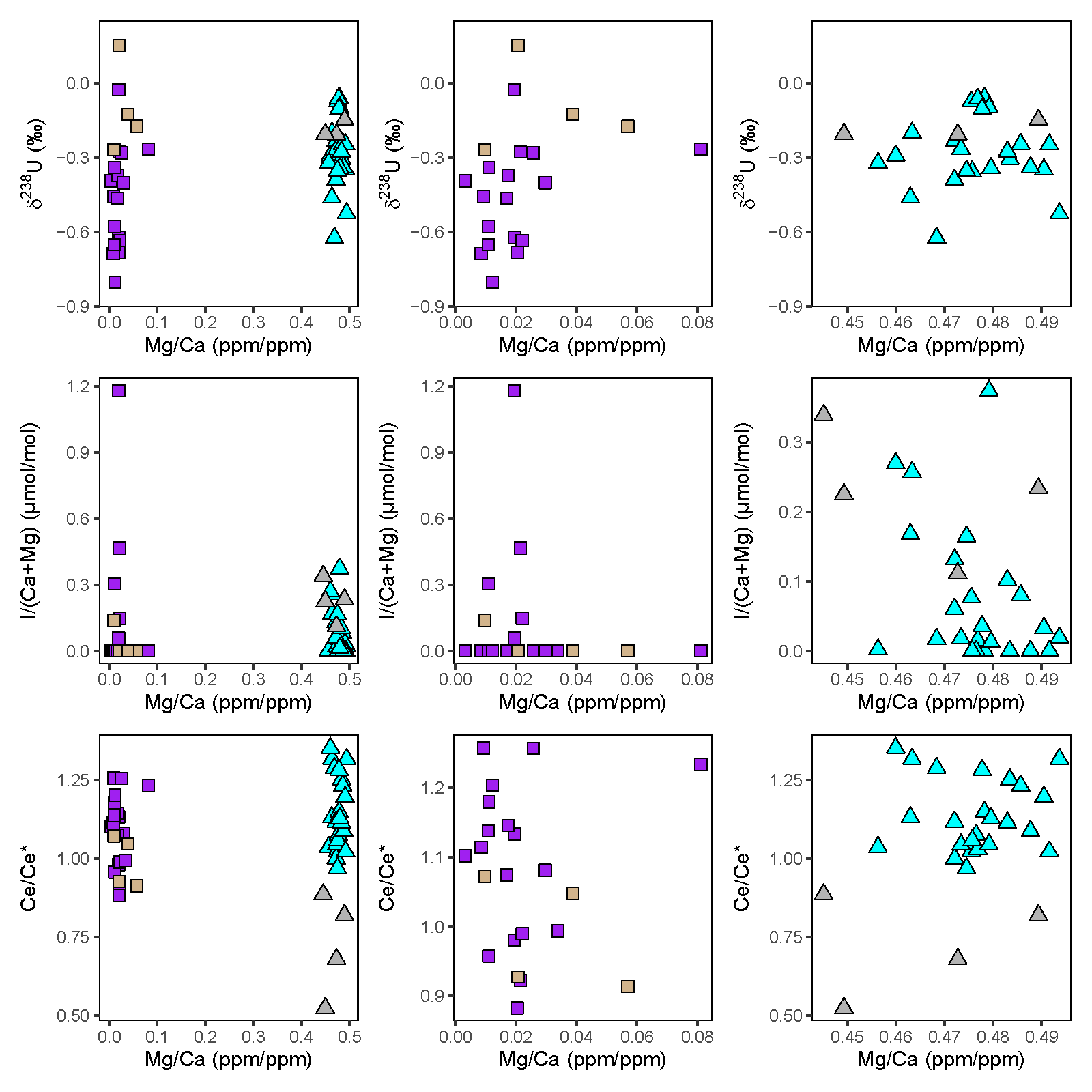


**Figure S8** – Crossplots of carbonate redox proxies (i.e., δ^238^U_carb_, Ce/Ce*, and I/(Ca+Mg)) versus Mg/Ca ratios from the Deep Spring Formation at Mount Dunfee (squares) and the La Ciénega Formation at Cerro Rajón (triangle; excluding two anomalously low δ^238^U_carb_ below −1‰ in the upper panel). Tan squares indicate the Dunfee Member and grey triangles indicate samples proximal (< 4 m) to interstratified metabasalt sills.


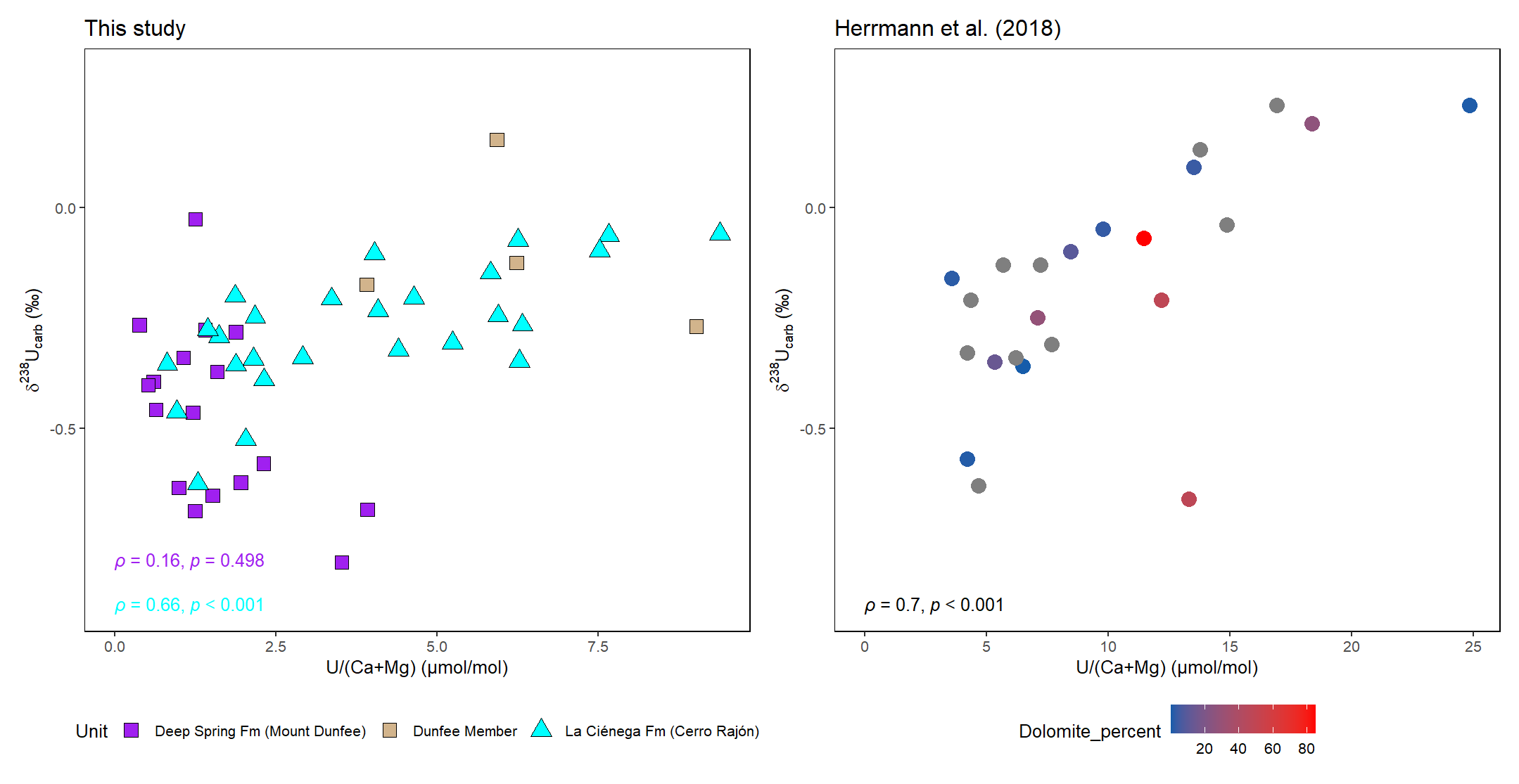


**Figure S9** – Crossplots of uranium isotope (δ^238^U_carb_) versus U/(Ca+Mg) ratio from the Deep Spring Formation at Mount Dunfee (purple squares = Esmeralda Member; tan squares = Dunfee Member), the La Ciénega Formation at Cerro Rajón (cyan triangles; excluding two anomalously low δ^238^U_carb_ below −1‰), and the Moosburg core from Southern Germany (circles; modified after Herrmann et al. (2018)). Spearman’s rank correlation statistics are shown as Spearman’s ρ and *p*-values and colored by study sections.


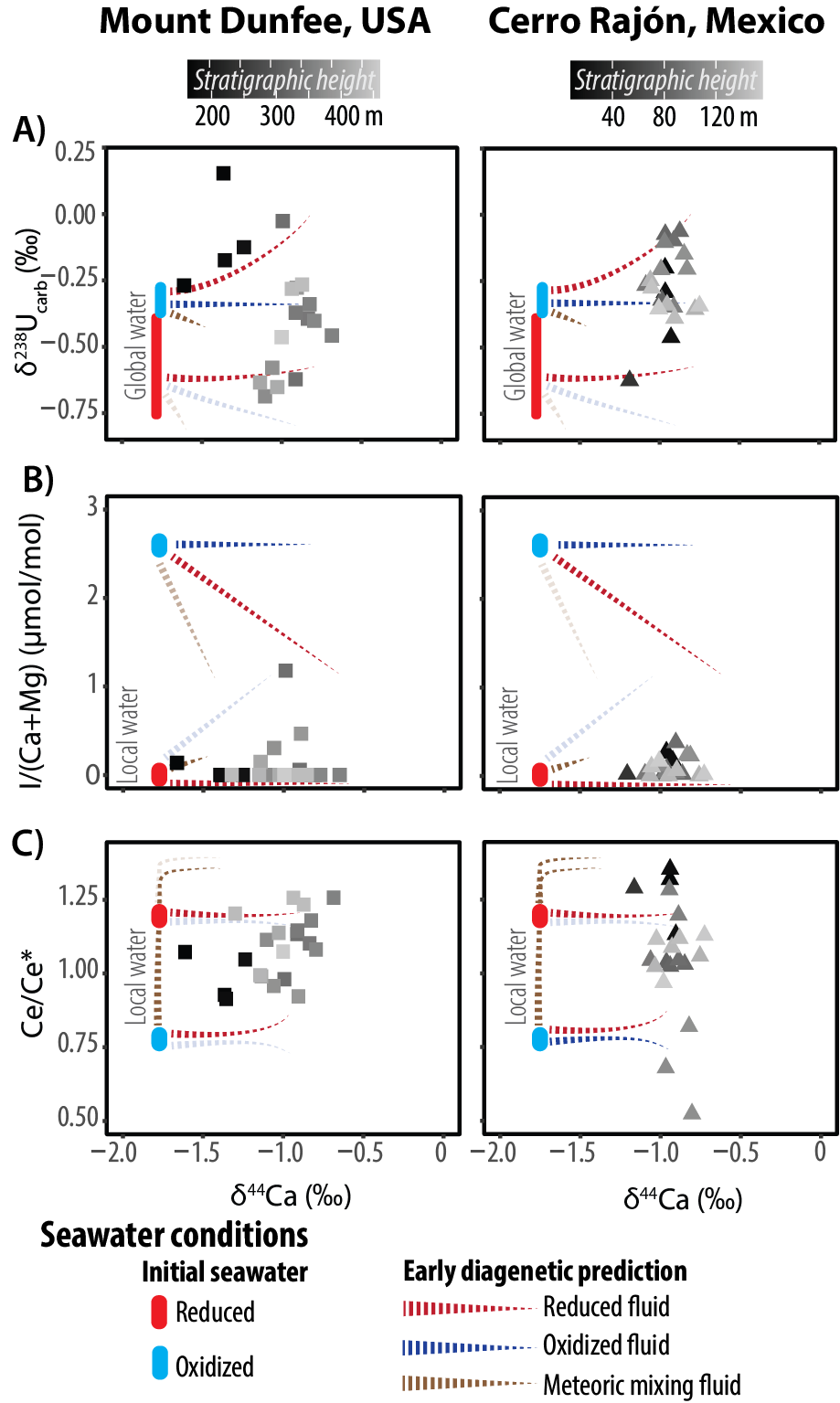


**Figure S10** – Crossplots of δ^44^Ca data (modified from Lonsdale et al., *accepted*) versus global and local seawater conditions of (A) δ^238^U_carb_ records, (B) I/(Ca+Mg) ratios, and (C) Ce anomalies from the Deep Spring Formation at Mount Dunfee (left panel) and the La Ciénega Formation at Cerro Rajón (right panel). Grayscale gradient corresponds to stratigraphic height. Colored rounded square and dashed arrows overlie predictions of initial seawater conditions (i.e., reduced vs oxidized water) and early diagenetic transformations via various fluids (i.e., reduced, oxidized, and meteoric fluids) after a diagenetic modeling study (Lau & Hardisty, 2022).


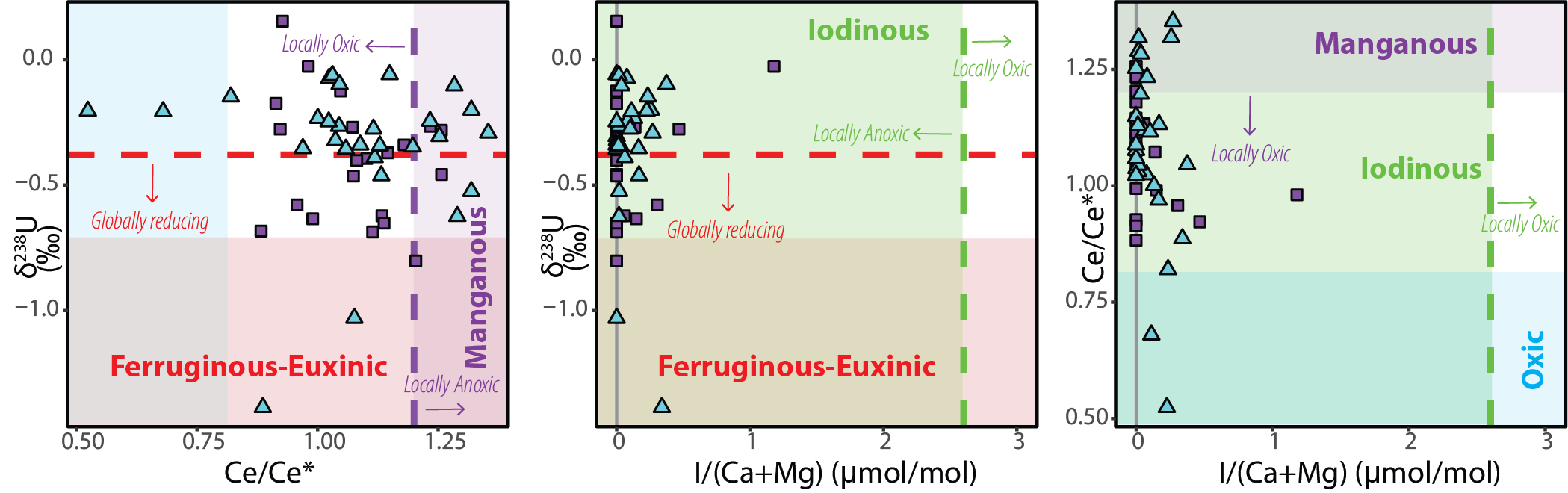


**Figure S11** – Crossplots of carbonate-based redox proxies (i.e., δ^238^U_carb_, Ce/Ce*, and I/(Ca+Mg)). Purple squares indicate the Deep Spring Formation and cyan triangles indicate the La Ciénega Formation at Cerro Rajón. Colored regions: interpretations of global/ local redox conditions, such as oxic, iodinous, manganous, and ferruginous–euxinic (also see Figure 2 and Figure 7). Dashed lines: red = modern global mean seawater δ^238^U (−0.379 ± 0.023‰; Kipp et al., 2022; Tissot & Dauphas, 2015); green = modern oxygenated seawater for I/(Ca+Mg) ratio (2.6 μmol/mol; Glock et al., 2014; Lu et al., 2016); purple = positive Ce anomaly (Ce/Ce* > 1.2; Tostevin, 2021).


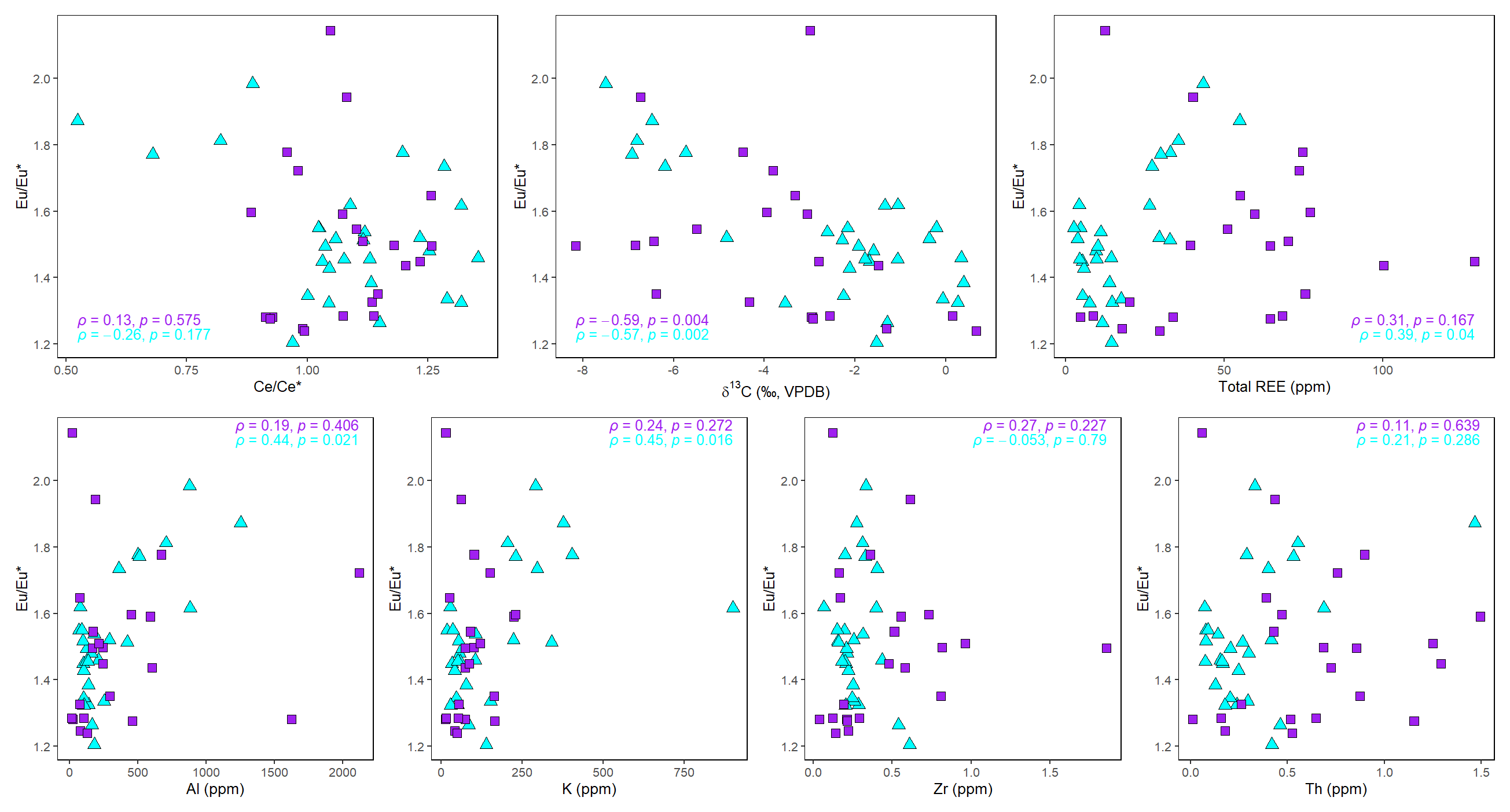


**Figure S12** – Crossplots of Eu anomaly (Eu/Eu*) versus Ce anomaly (Ce/Ce*), carbon isotope (δ^13^C), total rare earth element (upper panel) and clay contents, such as Al, K, Zr, and Th (lower panel) from the Deep Spring Formation at Mount Dunfee (purple squares) and the La Ciénega Formation at Cerro Rajón (cyan triangles). Spearman’s rank correlation statistics are shown (purple = Deep Spring Formation and cyan = Cerro Rajón) as Spearman’s ρ and *p*-values.

**References**

Ahm, A.-S. C., Bjerrum, C. J., Blättler, C. L., Swart, P. K., & Higgins, J. A. (2018). Quantifying early marine diagenesis in shallow-water carbonate sediments. *Geochimica et Cosmochimica Acta*, *236*, 140–159. https://doi.org/10.1016/j.gca.2018.02.042

Barrón-Díaz, A. J., Paz-Moreno, F. A., & Hagadorn, J. W. (2019). The Cerro Rajón Formation—A new lithostratigraphic unit proposed for a Cambrian (Terreneuvian) volcano-sedimentary succession from the Caborca region, northwest Mexico. *Journal of South American Earth Sciences*, *89*, 197–210. https://doi.org/10.1016/j.jsames.2018.11.003

Bau, M. (1991). Rare-earth element mobility during hydrothermal and metamorphic fluid-rock interaction and the significance of the oxidation state of europium. *Chemical Geology*, *93*(3), 219–230. https://doi.org/10.1016/0009-2541(91)90115-8

Bau, M., & Dulski, P. (1996). Distribution of yttrium and rare-earth elements in the Penge and Kuruman iron-formations, Transvaal Supergroup, South Africa. *Precambrian Research*, *79*(1–2), 37–55. https://doi.org/10.1016/0301-9268(95)00087-9

Bau, M., Koschinsky, A., Dulski, P., & Hein, J. R. (1996). Comparison of the partitioning behaviours of yttrium, rare earth elements, and titanium between hydrogenetic marine ferromanganese crusts and seawater. *Geochimica et Cosmochimica Acta*, *60*(10), 1709–1725. https://doi.org/10.1016/0016-7037(96)00063-4

Bau, M., Schmidt, K., Koschinsky, A., Hein, J., Kuhn, T., & Usui, A. (2014). Discriminating between different genetic types of marine ferro-manganese crusts and nodules based on rare earth elements and yttrium. *Chemical Geology*, *381*, 1–9. https://doi.org/10.1016/j.chemgeo.2014.05.004

Bond, G. C., Christie-Blick, N., Kominz, M. A., & Devlin, W. J. (1985). An early Cambrian rift to post-rift transition in the Cordillera of western North America. *Nature*, *315*(6022), 742–746. https://doi.org/10.1038/315742a0

Cao, C., Liu, X.-M., Bataille, C. P., & Liu, C. (2020). What do Ce anomalies in marine carbonates really mean? A perspective from leaching experiments. *Chemical Geology*, *532*, 119413. https://doi.org/10.1016/j.chemgeo.2019.119413

Chen, S., Gui, H., & Sun, L. (2014). Geochemical characteristics of REE in the Late Neo-proterozoic limestone from northern Anhui Province, China. *Chinese Journal of Geochemistry*, *33*(2), 187–193. https://doi.org/10.1007/s11631-014-0677-z

de Baar, H. J. W., German, C. R., Elderfield, H., & van Gaans, P. (1988). Rare earth element distributions in anoxic waters of the Cariaco Trench. *Geochimica et Cosmochimica Acta*, *52*(5), 1203–1219. https://doi.org/10.1016/0016-7037(88)90275-X

Dekov, V. M., Cuadros, J., Shanks, W. C., & Koski, R. A. (2008). Deposition of talc — kerolite–smectite — smectite at seafloor hydrothermal vent fields: Evidence from mineralogical, geochemical and oxygen isotope studies. *Chemical Geology*, *247*(1–2), 171–194. https://doi.org/10.1016/j.chemgeo.2007.10.022

Elderfield, H., & Greaves, M. J. (1982). The rare earth elements in seawater. *Nature*, *296*(5854), 214–219. https://doi.org/10.1038/296214a0

Farmer, G. L., Espinoza, G., Morales, M., Martin, M. W., & Bowring, S. A. (2001). Nd isotope constraints on sources of Neoproterozoic to Early Cambrian siliciclastic sedimentary rocks in northern Sonora. *Journal of South American Earth Sciences*, *14*(5), 437–446. https://doi.org/10.1016/S0895-9811(01)00053-0

Glock, N., Liebetrau, V., & Eisenhauer, A. (2014). I/Ca ratios in benthic foraminifera from the Peruvian oxygen minimum zone: Analytical methodology and evaluation as a proxy for redox conditions. *Biogeosciences*, *11*(23), 7077–7095. https://doi.org/10.5194/bg-11-7077-2014

Hardisty, D. S., Lu, Z., Bekker, A., Diamond, C. W., Gill, B. C., Jiang, G., Kah, L. C., Knoll, A. H., Loyd, S. J., Osburn, M. R., Planavsky, N. J., Wang, C., Zhou, X., & Lyons, T. W. (2017). Perspectives on Proterozoic surface ocean redox from iodine contents in ancient and recent carbonate. *Earth and Planetary Science Letters*, *463*, 159–170. https://doi.org/10.1016/j.epsl.2017.01.032

Hashim, M. S., Burke, J. E., Hardisty, D. S., & Kaczmarek, S. E. (2022). Iodine incorporation into dolomite: Experimental constraints and implications for the iodine redox proxy and Proterozoic Ocean. *Geochimica et Cosmochimica Acta*, *338*, 365–381. https://doi.org/10.1016/j.gca.2022.10.027

Herrmann, A. D., Gordon, G. W., & Anbar, A. D. (2018). Uranium isotope variations in a dolomitized Jurassic carbonate platform (Tithonian; Franconian Alb, Southern Germany). *Chemical Geology*, *497*, 41–53. https://doi.org/10.1016/j.chemgeo.2018.08.017

Higgins, J. A., Blättler, C. L., Lundstrom, E. A., Santiago-Ramos, D. P., Akhtar, A. A., Crüger Ahm, A.-S., Bialik, O., Holmden, C., Bradbury, H., Murray, S. T., & Swart, P. K. (2018). Mineralogy, early marine diagenesis, and the chemistry of shallow-water carbonate sediments. *Geochimica et Cosmochimica Acta*, *220*, 512–534. https://doi.org/10.1016/j.gca.2017.09.046

Hodgin, E. B., Nelson, L. L., Wall, C. J., Barrón-Díaz, A. J., Webb, L. C., Schmitz, M. D., Fike, D. A., Hagadorn, J. W., & Smith, E. F. (2021). A link between rift-related volcanism and end-Ediacaran extinction? Integrated chemostratigraphy, biostratigraphy, and U-Pb geochronology from Sonora, Mexico. *Geology*, *49*(2), 115–119. https://doi.org/10.1130/G47972.1

Hohl, S. V., Becker, H., Jiang, S.-Y., Ling, H.-F., Guo, Q., & Struck, U. (2017). Geochemistry of Ediacaran cap dolostones across the Yangtze Platform, South China: Implications for diagenetic modification and seawater chemistry in the aftermath of the Marinoan glaciation. *Journal of the Geological Society*, *174*(5), 893–912. https://doi.org/10.1144/jgs2016-145

Hu, M., Ngia, N. R., & Gao, D. (2019). Dolomitization and hydrotectonic model of burial dolomitization of the Furongian-Lower Ordovician carbonates in the Tazhong Uplift, central Tarim Basin, NW China: Implications from petrography and geochemistry. *Marine and Petroleum Geology*, *106*, 88–115. https://doi.org/10.1016/j.marpetgeo.2019.04.018

Kalderon-Asael, B., Katchinoff, J. A. R., Planavsky, N. J., Hood, A. V. S., Dellinger, M., Bellefroid, E. J., Jones, D. S., Hofmann, A., Ossa, F. O., Macdonald, F. A., Wang, C., Isson, T. T., Murphy, J. G., Higgins, J. A., West, A. J., Wallace, M. W., Asael, D., & Pogge Von Strandmann, P. A. E. (2021). A lithium-isotope perspective on the evolution of carbon and silicon cycles. *Nature*, *595*(7867), 394–398. https://doi.org/10.1038/s41586-021-03612-1

Kamber, B. S., Greig, A., & Collerson, K. D. (2005). A new estimate for the composition of weathered young upper continental crust from alluvial sediments, Queensland, Australia. *Geochimica et Cosmochimica Acta*, *69*(4), 1041–1058. https://doi.org/10.1016/j.gca.2004.08.020

Kamber, B. S., & Webb, G. E. (2001). The geochemistry of late Archaean microbial carbonate: Implications for ocean chemistry and continental erosion history. *Geochimica et Cosmochimica Acta*, *65*(15), 2509–2525. https://doi.org/10.1016/S0016-7037(01)00613-5

Kipp, M. A., Li, H., Ellwood, M. J., John, S. G., Middag, R., Adkins, J. F., & Tissot, F. L. H. (2022). 238U, 235U and 234U in seawater and deep-sea corals: A high-precision reappraisal. *Geochimica et Cosmochimica Acta*, *336*, 231–248. https://doi.org/10.1016/j.gca.2022.09.018

Lau, K. V., & Hardisty, D. S. (2022). Modeling the impacts of diagenesis on carbonate paleoredox proxies. *Geochimica et Cosmochimica Acta*, *337*, 123–139. https://doi.org/10.1016/j.gca.2022.09.021

Lee, S.-G., Chen, S., Lee, T. J., Kim, H., Lee, S. R., Choi, S. H., Ahn, U. S., & Lee, Y. S. (2025). Eu isotope fractionation and hydrothermal alteration. *Scientific Reports*, *15*(1), 28746. https://doi.org/10.1038/s41598-025-14613-9

Levy, M., & Christie-Blick, N. (1991). Tectonic subsidence of the early Paleozoic passive continental margin in eastern California and southern Nevada. *Geological Society of America Bulletin*, *103*(12), 1590–1606. https://doi.org/10.1130/0016-7606(1991)103%253C1590:TSOTEP%253E2.3.CO;2

Ling, H.-F., Chen, X., Li, D., Wang, D., Shields-Zhou, G. A., & Zhu, M. (2013). Cerium anomaly variations in Ediacaran–earliest Cambrian carbonates from the Yangtze Gorges area, South China: Implications for oxygenation of coeval shallow seawater. *Precambrian Research*, *225*, 110–127. https://doi.org/10.1016/j.precamres.2011.10.011

Loges, A., Wagner, T., Barth, M., Bau, M., Göb, S., & Markl, G. (2012). Negative Ce anomalies in Mn oxides: The role of Ce4+ mobility during water–mineral interaction. *Geochimica et Cosmochimica Acta*, *86*, 296–317. https://doi.org/10.1016/j.gca.2012.03.017

Lonsdale, M.C. (2025). REGIONAL PERSPECTIVES ON GLOBAL GEOBIOLOGICAL TRANSITIONS ACROSS THE NEOPROTEROZOIC ERA [Doctoral dissertation, Johns Hopkins University].

Lonsdale, M. C., Ahm, A. C., Nelson, L. L., Thompson, J., Higgins, J. A. & Smith, E. F. (accepted). Using calcium isotope to assess the influence of marine diagenesis at the Precambrian-Cambrian boundary in western Laurentia. *American Journal of Science*.

Lu, Z., Hoogakker, B. A. A., Hillenbrand, C.-D., Zhou, X., Thomas, E., Gutchess, K. M., Lu, W., Jones, L., & Rickaby, R. E. M. (2016). Oxygen depletion recorded in upper waters of the glacial Southern Ocean. *Nature Communications*, *7*(1), 11146. https://doi.org/10.1038/ncomms11146

Meyer, E. E., Quicksall, A. N., Landis, J. D., Link, P. K., & Bostick, B. C. (2012). Trace and rare earth elemental investigation of a Sturtian cap carbonate, Pocatello, Idaho: Evidence for ocean redox conditions before and during carbonate deposition. *Precambrian Research*, *192–195*, 89–106. https://doi.org/10.1016/j.precamres.2011.09.015

Mitra, A., Elderfield, H., & Greaves, M. J. (1994). Rare earth elements in submarine hydrothermal fluids and plumes from the Mid-Atlantic Ridge. *Marine Chemistry*, *46*(3), 217–235. https://doi.org/10.1016/0304-4203(94)90079-5

Nothdurft, L. D., Webb, G. E., & Kamber, B. S. (2004). Rare earth element geochemistry of Late Devonian reefal carbonates, Canning Basin, Western Australia: Confirmation of a seawater REE proxy in ancient limestones. *Geochimica et Cosmochimica Acta*, *68*(2), 263–283. https://doi.org/10.1016/S0016-7037(03)00422-8

Nozaki, Y., Lerche, D., Alibo, D. S., & Snidvongs, A. (2000). The estuarine geochemistry of rare earth elements and indium in the Chao Phraya River, Thailand. *Geochimica et Cosmochimica Acta*, *64*(23), 3983–3994. https://doi.org/10.1016/S0016-7037(00)00473-7

Olivarez, A. M., & Owen, R. M. (1991). The europium anomaly of seawater: Implications for fluvial versus hydrothermal REE inputs to the oceans. *Chemical Geology*, *92*(4), 317–328. https://doi.org/10.1016/0009-2541(91)90076-4

Schijf, J., De Baar, H. J. W., & Millero, F. J. (1995). Vertical distributions and speciation of dissolved rare earth elements in the anoxic brines of Bannock Basin, eastern Mediterranean Sea. *Geochimica et Cosmochimica Acta*, *59*(16), 3285–3299. https://doi.org/10.1016/0016-7037(95)00219-P

Seto, M., & Akagi, T. (2008). Chemical condition for the appearance of a negative Ce anomaly in stream waters and groundwaters. *GEOCHEMICAL JOURNAL*, *42*(4), 371–380. https://doi.org/10.2343/geochemj.42.371

Shields, G., & Stille, P. (2001). Diagenetic constraints on the use of cerium anomalies as palaeoseawater redox proxies: An isotopic and REE study of Cambrian phosphorites. *Chemical Geology*, *175*(1–2), 29–48. https://doi.org/10.1016/S0009-2541(00)00362-4

Smith, E. F., Nelson, L. L., Strange, M. A., Eyster, A. E., Rowland, S. M., Schrag, D. P., & Macdonald, F. A. (2016). The end of the Ediacaran: Two new exceptionally preserved body fossil assemblages from Mount Dunfee, Nevada, USA. *Geology*, *44*(11), 911–914. https://doi.org/10.1130/G38157.1

Sverjensky, D. A. (1984). Europium redox equilibria in aqueous solution. *Earth and Planetary Science Letters*, *67*(1), 70–78. https://doi.org/10.1016/0012-821X(84)90039-6

Taylor, S. R., & McLennan, S. M. (1985). The Continental Crust: Its Composition and Evolution. *Blackwell Scientific*, *122*(6), 312. https://doi.org/10.1017/S0016756800032167

Tissot, F. L. H., & Dauphas, N. (2015). Uranium isotopic compositions of the crust and ocean: Age corrections, U budget and global extent of modern anoxia. *Geochimica et Cosmochimica Acta*, *167*, 113–143. https://doi.org/10.1016/j.gca.2015.06.034

Tostevin, R. (2021). *Cerium Anomalies and Paleoredox* (1st ed.). Cambridge University Press. https://doi.org/10.1017/9781108847223

Tostevin, R., Shields, G. A., Tarbuck, G. M., He, T., Clarkson, M. O., & Wood, R. A. (2016a). Effective use of cerium anomalies as a redox proxy in carbonate-dominated marine settings. *Chemical Geology*, *438*, 146–162. https://doi.org/10.1016/j.chemgeo.2016.06.027

Tostevin, R., Shields, G. A., Tarbuck, G. M., He, T., Clarkson, M. O., & Wood, R. A. (2016b). Effective use of cerium anomalies as a redox proxy in carbonate-dominated marine settings. *Chemical Geology*, *438*, 146–162. https://doi.org/10.1016/j.chemgeo.2016.06.027

Tostevin, R., Wood, R. A., Shields, G. A., Poulton, S. W., Guilbaud, R., Bowyer, F., Penny, A. M., He, T., Curtis, A., Hoffmann, K. H., & Clarkson, M. O. (2016c). Low-oxygen waters limited habitable space for early animals. *Nature Communications*, *7*(1), 12818. https://doi.org/10.1038/ncomms12818

Zhang, K., & Shields, G. A. (2023). Early diagenetic mobilization of rare earth elements and implications for the Ce anomaly as a redox proxy. *Chemical Geology*, *635*, 121619. https://doi.org/10.1016/j.chemgeo.2023.121619
